# Composition-controlled graphene–ZnO–SiC–reinforced aluminum matrix composites: mechanical ranking and minimum quantity lubrication-assisted turning optimization

**DOI:** 10.1039/d6ra04327e

**Published:** 2026-11-02

**Authors:** Md. Sazzad Hossain Ador, Md. Mahraj Uddin, Md Zillur Rahman

**Affiliations:** a Department of Industrial and Production Engineering, Ahsanullah University of Science and Technology Dhaka 1208 Bangladesh; b Department of Industrial and Production Engineering, Bangladesh University of Engineering and Technology Dhaka 1000 Bangladesh mahrajipesust12@gmail.com; c Department of Mechanical Engineering, Ahsanullah University of Science and Technology Dhaka 1208 Bangladesh md.zillur.rahman.phd@gmail.com

## Abstract

Lightweight aluminum metal matrix composites that combine high mechanical performance with reliable machinability are essential for advanced aerospace, automotive, and precision manufacturing applications. However, simultaneously optimizing strength, damage tolerance, surface integrity, tool wear, and sustainable machining remains challenging, particularly in tri-hybrid systems containing ceramic, carbonaceous, and oxide reinforcements. This study develops composition-controlled graphene–zinc oxide–silicon carbide (Gr–ZnO–SiC)–reinforced aluminum metal matrix composites (Al-MMCs) through a multi-stage stir-casting route, with the Al matrix fixed at 90 wt% and the total reinforcement content maintained at 10 wt%. The internal reinforcement fraction was systematically varied by increasing Gr and ZnO from 1 to 4 wt% each while reducing SiC from 8 to 2 wt%, enabling the identification of a mechanically balanced composition rather than a single-property optimum. Among the fabricated composites, the C3 composition containing 2 wt% Gr, 2 wt% ZnO, and 6 wt% SiC showed the best overall response, achieving a tensile strength of 201.5 MPa, flexural strength of 283.3 MPa, impact strength of 43.3 kJ m^−2^, and hardness of 69.3 HV. Independent TOPSIS and PCA rankings confirmed C3 as the optimum composition, with a closeness coefficient of 0.967 and a PC1 score of 2.133. FESEM and fractographic analyses revealed a compact microstructure with no large interfacial gaps and a mixed ductile–quasi-cleavage fracture response, indicating effective load transfer, crack deflection, and resistance to localized damage. The optimized C3 composite was subsequently evaluated under dry and minimum-quantity lubrication turning conditions. Relative to dry machining, MQL reduced surface roughness by 43.31%, cutting force by 24.19%, cutting temperature by 8.62%, and chip reduction coefficient by 3.62%, while limiting flank wear from 0.31 to 0.21 mm after 30 min of cutting. Response surface models showed high predictive reliability with no significant lack of fit, and multi-response optimization identified stable MQL-assisted machining conditions of 150 m min^−1^ cutting speed, 0.16 mm per rev feed rate, and 1.0 mm depth of cut, yielding *R*_a_ = 1.468 µm, *F*_c_ = 137.604 N, *ξ* = 1.702, *T* = 284.275 °C, and a desirability of 0.897. These findings establish a composition-processing-machining framework for developing damage-tolerant Al-MMCs with balanced structural performance and sustainable machinability.

## Abstract 2

At a fixed 10 wt% reinforcement, 2Gr–2ZnO–6SiC/Al showed the best overall mechanical performance, reaching 201.5 MPa tensile strength and 283.3 MPa flexural strength; MQL reduced roughness by 43% and cutting force by 24% *versus* dry turning.

## Graphical abstract

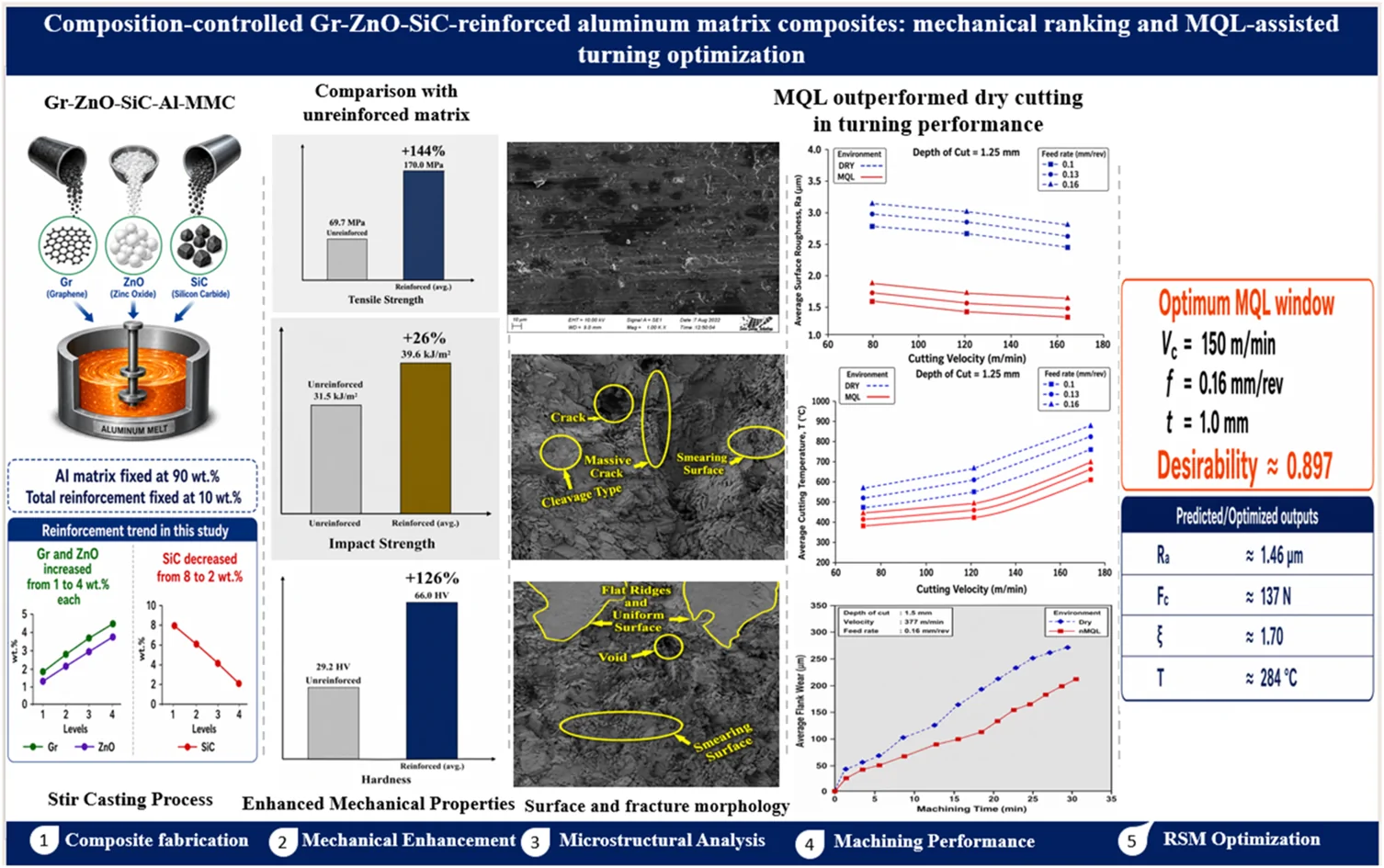

## 1 Introduction

Structural components used in aerospace, automotive, and transportation applications require materials that combine high specific strength, wear resistance, thermal stability, and reliable machinability while maintaining low component weight. Aluminum (Al) metal matrix composites (Al-MMCs) are strong candidates because they combine the low density, castability, and corrosion resistance of Al with the stiffness, hardness, and tribological resistance provided by particulate reinforcements.^1,2^ However, unreinforced Al alloys generally show limited hardness, poor wear resistance, and inadequate load-bearing capacity under severe contact, sliding, or cyclic loading conditions. These limitations restrict their use in tribologically demanding components such as pistons, brake rotors, structural frames, and precision-machined parts.^3,4^ Incorporating micro- and nanoscale reinforcements into the Al matrix can overcome these deficiencies through load transfer, dislocation strengthening, grain refinement, and improved matrix–reinforcement bonding, enhancing mechanical and tribological performance without a substantial weight penalty.^1,3,4^

Reinforcement type significantly determines the performance of Al-MMCs, as each reinforcement contributes through distinct strengthening and damage-control mechanisms. Silicon carbide (SiC) is one of the most widely used ceramic reinforcements in Al-MMCs. Previous studies have reported that 10 to 15 wt% SiC can increase tensile strength by about 15% and hardness by about 10% compared with unreinforced Al alloys, mainly through load transfer and dislocation-based strengthening.^5^ However, the abrasive nature of SiC may promote tool wear during machining, and particle clustering at higher contents can reduce processing reliability and surface quality.^5,6^ Graphene (Gr) exhibits exceptional intrinsic tensile strength (∼130 GPa) and stiffness (∼1 TPa), along with solid-lubricating behavior arising from its layered hexagonal lattice structure, enabling load transfer, crack deflection, dislocation obstruction, and reduced interfacial friction when uniformly dispersed.^7,8^ Even at low contents, Gr can improve tribological behavior and contribute to toughening, thereby partially compensating for the brittleness introduced by hard ceramic particles.^8,9^ Zinc oxide (ZnO) nanoparticles further contribute through oxide-assisted strengthening, grain-boundary pinning, and improved interfacial bonding. An addition of 2 wt% ZnO has been reported to increase indentation hardness by about 28% in Al matrices, while also supporting more uniform stress transfer and microstructural stability in multiphase composites.^10,11^ Therefore, combining SiC, Gr, and ZnO is expected to offer complementary benefits: SiC provides load-bearing capacity and wear resistance, Gr improves lubrication and crack resistance, and ZnO supports interfacial bonding and microstructural refinement.^2,12,13^

Micro- and nanoscale reinforcements can be incorporated into Al matrices through liquid-state, semi-solid, and solid-state routes, including stir casting, squeeze casting or pressure infiltration, compocasting, powder metallurgy (PM), and friction stir processing (FSP).^1,3,14^ Stir casting is attractive for bulk Al-MMC production because of its relatively simple equipment, low cost, compatibility with conventional foundry practice, and scalability.^14,15^ Its main limitations include poor particle wettability, agglomeration, flotation or sedimentation arising from density differences, gas entrapment, porosity, and possible interfacial reactions, particularly with nanoscale reinforcements.^14–16^ Pressure-assisted casting can improve densification but requires additional equipment and tooling, whereas PM offers better control of particle distribution at the expense of additional processing steps and restrictions on component size and geometry. FSP avoids several melt-related difficulties but is generally confined to the processed region and is sensitive to tool geometry and processing conditions.^1,3^ These challenges are particularly relevant to the present Gr–ZnO–SiC system because the three reinforcements differ in density, morphology, and wetting behavior, while Gr is prone to agglomeration and poor wettability by molten Al.^15^ A controlled multi-stage stir-casting route was therefore selected to retain the economic and scale-up advantages of liquid processing while reducing the dispersion and segregation difficulties associated with dissimilar reinforcements.^14,15,17^

Mechanical performance alone is insufficient to assess the practical value of Al-MMCs, as reinforced composites must also be machined into components with dimensional accuracy and acceptable surface integrity. The same hard ceramic particles that improve hardness and wear resistance during service can accelerate tool abrasion, increase cutting force and temperature, and deteriorate machined surface quality during turning.^13,18^ Turning is particularly important for cylindrical and rotational Al-MMC components and directly assesses machinability responses, including surface roughness (*R*_a_), cutting force (*F*_c_), cutting temperature (*T*), chip reduction coefficient, and tool flank wear (*V*_B_).^19,20^ Minimum quantity lubrication (MQL) has attracted considerable attention as a sustainable alternative to dry and flood machining because it delivers a fine lubricant mist directly to the cutting zone, reducing friction, heat generation, adhesion, and tool wear while limiting coolant consumption and disposal concerns.^21,22^ Khandey *et al.*^21^ reported that MQL turning of Al-MMCs reduced cutting force by 17% and 50% compared with dry and wet machining, respectively, while improving surface finish. Kannan *et al.*^22^ similarly found that MQL turning of Al hybrid nanocomposites resulted in lower cutting forces, reduced tool wear, and better surface quality than dry cutting. These findings show that lubrication condition and reinforcement composition should be evaluated together when assessing the manufacturability of a new hybrid composite.

Although many studies have examined single-reinforced and dual-reinforced Al-MMCs, integrated studies on tri-hybrid Al-MMCs containing ceramic, carbonaceous, and oxide reinforcements remain limited.^1,2,6,20^ In particular, few reports have systematically varied the relative fractions of Gr, ZnO, and SiC at a fixed total reinforcement content and then linked the resulting mechanical ranking with dry and MQL-assisted turning behavior. This gap is important because the composition that maximizes hardness may not provide the best balance of tensile strength, flexural strength, impact resistance, surface quality, cutting force, cutting temperature, chip deformation, and tool wear. A multi-response framework is therefore required to identify a balanced composite rather than a single-property optimum.

This study addresses this need by fabricating, characterizing, and machining stir-cast Al-based tri-hybrid composites reinforced with Gr, ZnO, and SiC. The Al matrix was fixed at 90 wt%, while the total reinforcement content was maintained at 10 wt%. The Gr and ZnO contents were increased from 1 to 4 wt% each, while the SiC content was reduced from 8 to 2 wt%, enabling a systematic assessment of reinforcement ratio effects. The objectives were to: (i) evaluate tensile strength, flexural strength, impact strength, and Vickers hardness; (ii) examine microstructure, fracture morphology, and machined surface characteristics using field-emission scanning electron microscopy (FESEM); (iii) compare dry and MQL turning performance based on surface roughness, cutting force, chip reduction coefficient, cutting temperature, and flank wear; (iv) rank the fabricated composites using the Technique for Order Preference by Similarity to Ideal Solution (TOPSIS) and Principal Component Analysis (PCA); and (v) model and optimize machining parameters, including cutting speed, feed rate, and depth of cut, using Response Surface Methodology (RSM). The findings provide practical guidance on selecting reinforcement ratios and turning conditions for lightweight structural and tribological components that require strength, wear resistance, surface integrity, and manufacturing efficiency.

## 2 Materials and methods

### 2.1 Materials and reinforcement selection

Commercial Al bars were used as the matrix, while Gr, ZnO, and SiC powders were used as reinforcements. Al was selected for its low density, castability, ductility, and corrosion resistance. However, unreinforced Al has limited hardness, wear resistance, and load-bearing capacity, which justifies reinforcement with ceramic and carbonaceous phases.^4,14^ Gr was incorporated because of its high intrinsic stiffness and strength, which can support load transfer and crack-deflection mechanisms when dispersion and interfacial bonding are sufficient.^7,23^ ZnO was selected as an oxide reinforcement to improve hardness and resistance to local plastic deformation, while SiC was used as the principal ceramic load-bearing phase because of its high hardness and thermal stability.^24^ The ternary Gr–ZnO–SiC system was therefore manufactured to combine the toughening and lubricating contribution of Gr, the oxide-assisted strengthening effect of ZnO, and the indentation resistance of SiC. The chemical composition, physical form, particle-size specification, manufacturer-reported purity or assay, and source of the materials are summarized in Table 1. Fig. 1 shows representative photographs of the Al matrix and reinforcement materials.

**Table 1:** Chemical composition, particle specification, manufacturer-reported purity or assay, and source of the matrix and reinforcement materials.^25–28^

Material	Composition	Form/particle specification	Manufacturer-reported purity/assay	Source
Al	Al 99.052, Si 0.614, Fe 0.323, Zn 0.008, Cu 0.002, Pb 0.0009, Zr 0.0007 wt%	Commercial bar/ingot	99.052 wt% Al	BITAC, Bangladesh
Gr nanoplatelets	C	Powder; large lateral dimensions	>95 wt% C; <2 wt% O	Sigma-Aldrich, Cat. No. 806668
ZnO	ZnO	Nanopowder; ≤50 nm	∼80 wt% Zn, Zn-basis assay	Sigma-Aldrich, Cat. No. 544906
SiC	SiC	Powder; particle size 0.1–1 µm	99%	Goodfellow/Sigma-Aldrich, GF32877445

**Fig. 1:**
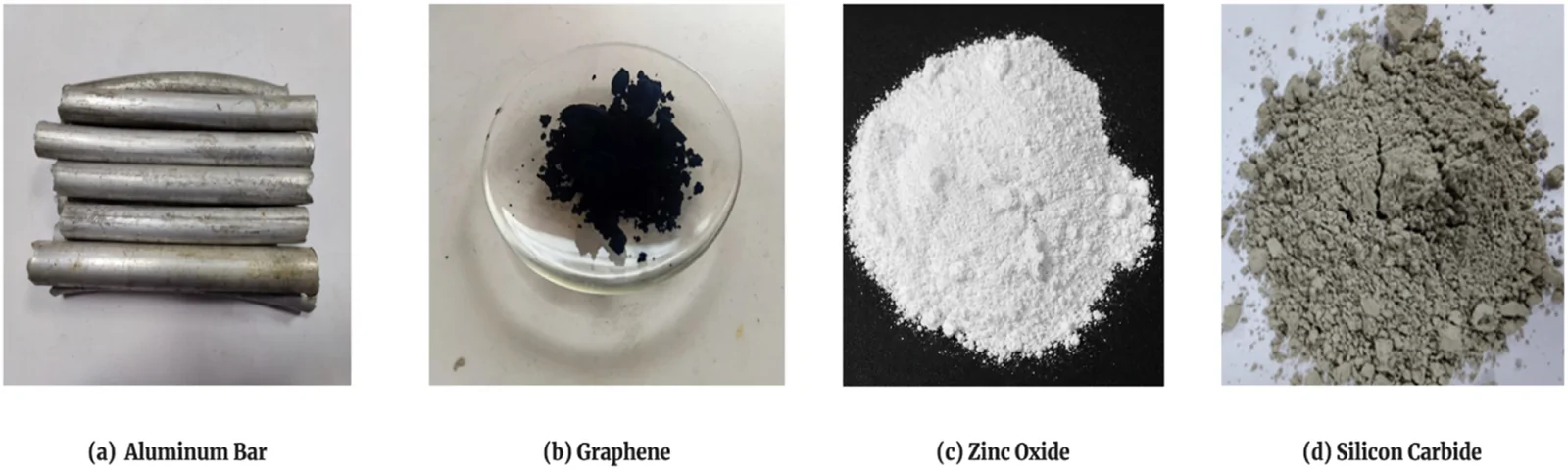
Raw materials used for composite fabrication: (a) Al matrix, (b) Gr nanoplatelets, (c) zinc oxide (ZnO), and (d) silicon carbide (SiC).

To isolate the effect of reinforcement partitioning, the Al matrix was fixed at 90 wt% and the total reinforcement content at 10 wt% for all composites. Gr and ZnO were increased from 1 to 4 wt% each, while SiC was reduced from 8 to 2 wt%. This range was selected because stir-cast Al-MMCs are sensitive to reinforcement clustering, porosity, and loss of melt fluidity at higher particle contents.^4,14^ Gr additions were limited to 1–4 wt% because solidification-processed Al–Gr composites often show agglomeration and porosity-related property loss above approximately 0.5–3 wt%.^16^ ZnO was restricted to 1–4 wt% because ZnO nanoparticle-reinforced metal matrix composites commonly show strengthening at moderate contents but may lose ductility when oxide clustering increases.^24^ SiC was retained within 2–8 wt% to maintain ceramic strengthening while preserving castability, as similar SiC ranges have been used for stir-cast Al2024/SiC_p_ composites.^29^ The selected composition range, therefore, balances strengthening efficiency, dispersion control, and casting feasibility. The composition details and nomenclature of the fabricated samples are summarized in Table 2.

**Table 2:** Composition and nomenclature of the fabricated samples

Sample code	Gr (wt%)	ZnO (wt%)	SiC (wt%)	Al (wt%)	Nomenclature
C1	0	0	0	100	100Al control
C2	1	1	8	90	1Gr–1ZnO–8SiC–90Al
C3	2	2	6	90	2Gr–2ZnO–6SiC–90Al
C4	3	3	4	90	3Gr–3ZnO–4SiC–90Al
C5	4	4	2	90	4Gr–4ZnO–2SiC–90Al

### 2.2 Composite fabrication

The SiC–ZnO–Gr–reinforced Al-MMCs were fabricated using a controlled multi-stage stir-casting route. The setup consisted of an electric resistance furnace, a graphite crucible, and a mechanically driven stirrer. Before melting, the crucible was preheated at 165 °C for 30 min to remove moisture and reduce gas-related casting defects. The Al bar was then charged into the crucible and melted at approximately 750–800 °C to provide sufficient melt fluidity for reinforcement incorporation. SiC and ZnO powders were preheated at 350 °C for 30 min to remove adsorbed moisture and improve their incorporation into the molten Al, whereas Gr was dried under vacuum at 100 °C for 2 h to minimize oxidation and structural degradation. The reinforcement-containing melt was stirred for 10 min, followed by an additional 5 min at 800 rpm to improve convective mixing, reduce particle agglomeration, and limit macroscopic segregation. This stirring condition was intended to promote reinforcement dispersion but does not ensure complete homogeneity throughout the molten composite. Because Gr, ZnO, and SiC differ in density and wettability with molten Al, localized flotation, sedimentation, or accumulation near the crucible wall may still occur during liquid-state processing. To limit these effects, the reinforcements were preheated or dried, added gradually, mixed continuously, and the slurry was poured into the preheated molds immediately after stirring was complete. The overall fabrication sequence for the Gr–ZnO–SiC–Al composites is shown in Fig. 2.

**Fig. 2:**
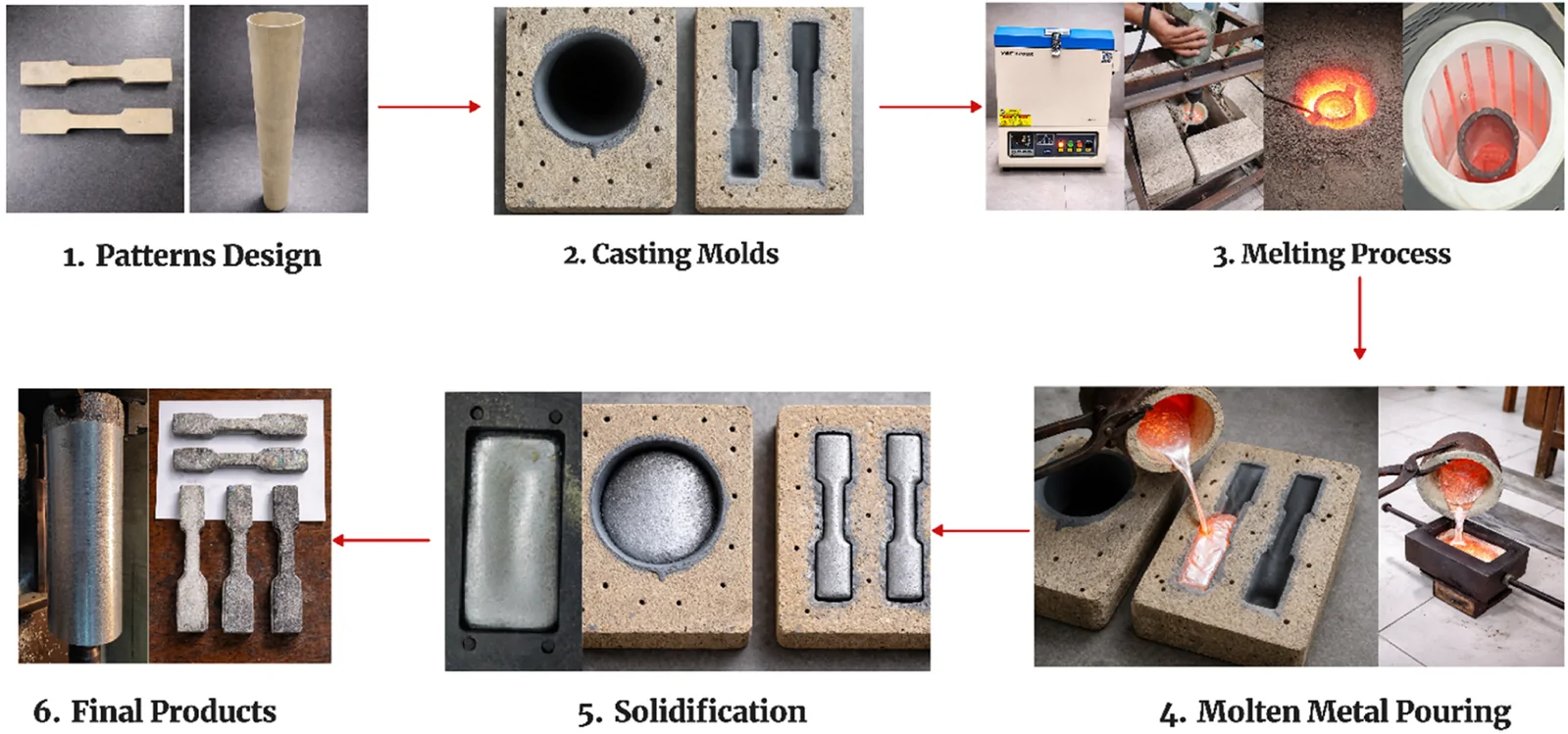
Fabrication sequence for the Gr–ZnO–SiC–Al composites.

### 2.3 Experimental framework

#### 2.3.1 Tensile testing

Tensile properties of the Al-MMC specimens were determined in accordance with ASTM B557M-15.^30^ Standard flat dog-bone specimens were precision-machined with a 50 mm gauge length and a 12.5 mm reduced-section width. The tests were performed using the Universal Testing Machine (UTM; H10KS, Hounsfield/Tinius Olsen Ltd, Surrey, United Kingdom) equipped with a 100 kN load cell. Loading was applied under displacement control at an initial crosshead speed of 0.5 mm min^−1^ up to the yield region, followed by 5 mm min^−1^ until specimen fracture. For each formulation, at least three independent specimens were tested, and the results are reported as mean ± standard deviation (SD).

#### 2.3.2 Flexural testing

Flexural testing of the Al-MMC specimens was performed using a three-point bending configuration on a universal testing machine (UTM; H10KS, Hounsfield/Tinius Olsen Ltd, Surrey, United Kingdom), following the test procedure and flexural strength calculation method prescribed in ASTM E290-22.^31^ Rectangular specimens with dimensions of 100 mm × 20 mm × 10 mm were prepared, maintaining a width-to-thickness ratio of 2 : 1. All specimen edges were smoothed prior to testing to minimize premature edge-initiated cracking. The support span length, crosshead displacement rate, and loading configuration were set in accordance with the standard. Flexural strength (*σ*_f_) was calculated using the standard three-point bending formula:1
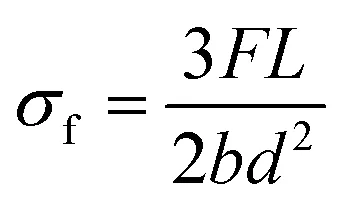
where *F* is the maximum applied load, *L* is the support span, *b* is the specimen width, and *d* is the specimen thickness.

#### 2.3.3 Impact testing

Charpy impact testing was performed in accordance with ASTM E23-25 (ref. 32) using an SI Series impact tester (Shimadzu Corporation, Kyoto, Japan). Specimens measured 55 mm × 10 mm × 10 mm and contained a 45° V-notch with 2 mm depth and 0.25 mm notch-root radius. The energy absorbed during the fracture was recorded from the instrument scale.

#### 2.3.4 Hardness testing

Micro-Vickers hardness was measured in accordance with ASTM E384-22 (ref. 33) using an HMV-G microhardness tester (Shimadzu Corporation, Kyoto, Japan). Polished specimens of 10 mm × 10 mm × 5 mm were prepared by standard metallographic grinding and polishing. Indentations were made using a Vickers diamond indenter under a 500-gf load with a 15 s dwell time. At least five measurements were taken at different locations on each specimen, and the mean value was reported.

#### 2.3.5 FESEM analysis

Field-emission scanning electron microscopy (FESEM; Philips/FEI XL30 SEM, Netherlands) was performed to examine the surface morphology and microstructural features of the Al-MMC specimens. Before FESEM observation, representative Al-MMC specimens were sectioned, ground with successive grades of silicon carbide abrasive papers, polished to obtain a smooth surface, and cleaned with acetone to remove surface contaminants. The samples were then dried and mounted on Al stubs using conductive carbon tape. For microstructural examination, polished surfaces were used, while fractured or tested surfaces were observed directly to preserve failure-related features. FESEM images were captured at different magnifications to evaluate reinforcement distribution, interfacial characteristics, porosity, and fracture behavior.

#### 2.3.6 MQL-assisted turning setup

Turning experiments were performed on a 7.5 kW conventional horizontal lathe (CA6140, China) under dry and minimum quantity lubrication (MQL) conditions. An uncoated carbide insert (ISO SNMG 120404) was mounted on a PSBNR 2525 M12 tool holder (WIDIA). The tool geometry consisted of a 6° side cutting-edge angle, 6° end cutting-edge angle, −6° side rake angle, and −6° back rake angle. An in-house-built MQL system supplied lubricant from a 2 L stainless-steel tank pressurized to approximately 7 bar. The lubricant was delivered to the cutting zone through a flexible line and applicator. Fig. 3 shows the MQL setup used for turning experiments.

**Fig. 3:**
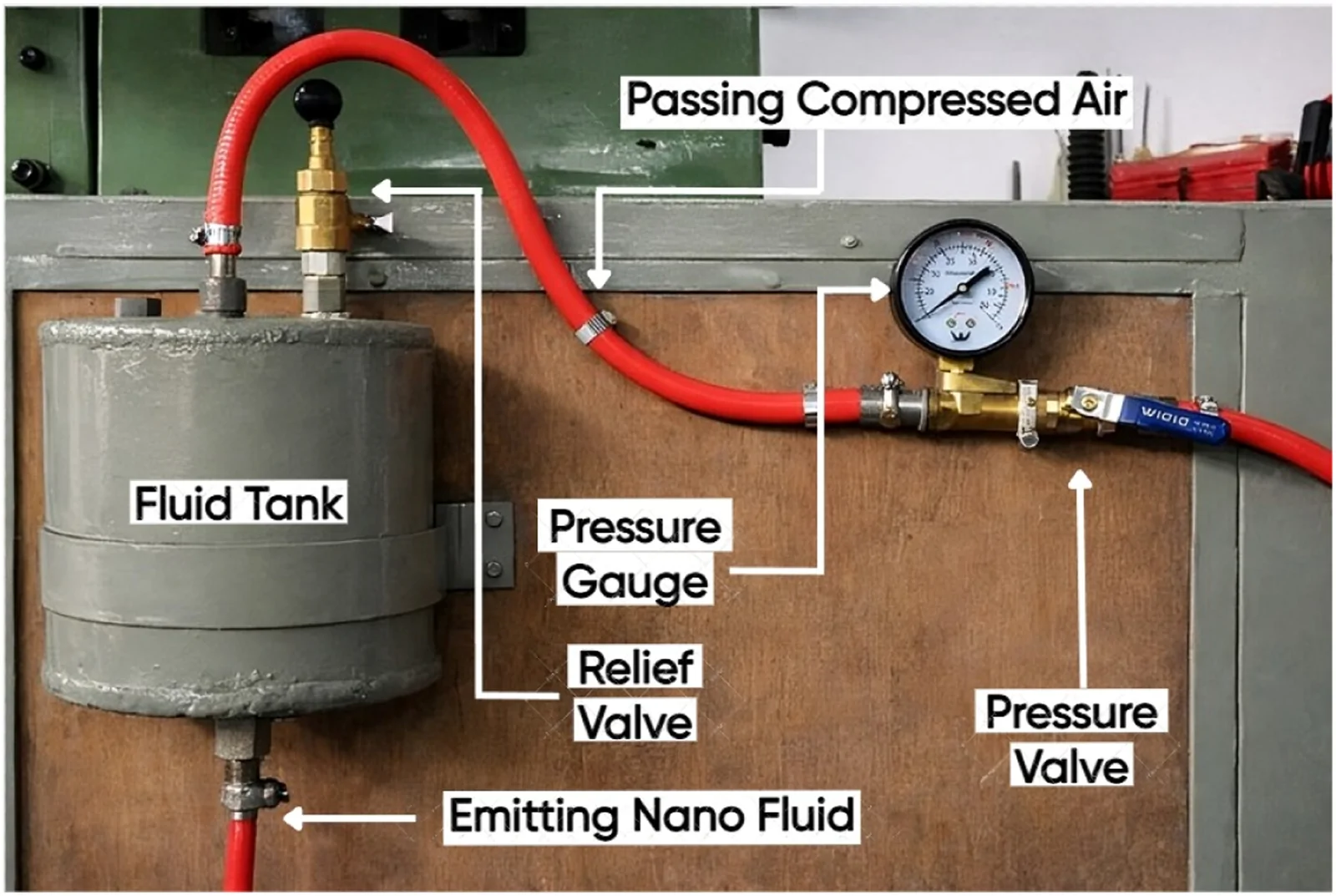
MQL setup used for turning experiments.

#### 2.3.7 Machining response evaluation and instrumentation

Machining performance was evaluated using cutting speed (*V*_c_), feed rate (*f*), depth of cut (*t*), and cutting environment as the input variables. These factors were selected because they directly influence chip formation, tool–chip contact, cutting load, frictional heat generation, surface quality, and tool wear during turning of particle-reinforced Al-MMCs.^19,21,22,34^ Cutting speed affects shear conditions, built-up-edge formation, contact time, and cutting temperature; feed rate governs uncut-chip thickness, feed marks, cutting force, and surface roughness; and depth of cut determines the engaged cutting area and associated mechanical and thermal loads. The cutting environment was evaluated under dry and MQL conditions to assess the influence of localized lubrication and friction reduction. These variables were subsequently included in the RSM to evaluate their individual, interaction, and quadratic effects.

Surface roughness was measured after each run using a portable roughness tester (Surf test SJ-210, Model 178-561-12A, Japan). Cutting temperature was measured using a calibrated tool–work thermocouple connected to a digital data acquisition unit. Cutting-force components (*F*_*x*_, *F*_*y*_, and *F*_*z*_) were recorded using a calibrated three-component piezoelectric dynamometer (Kistler 9129AA, Switzerland).

### 2.4 Statistical and optimization methods

#### 2.4.1 TOPSIS and PCA ranking procedure

TOPSIS and PCA were applied to evaluate and rank the fabricated composites based on their mechanical properties, namely tensile, flexural, and impact strengths, and hardness. All four responses were treated as beneficial criteria, where higher values indicate better mechanical performance. TOPSIS was used for multi-property material selection by ranking alternatives based on their closeness to the positive ideal solution and their distance from the negative ideal solution.^35^ PCA was employed as a complementary multivariate ranking method to summarize the correlated mechanical responses and compare the composition ranking obtained from TOPSIS.^36^

For the TOPSIS analysis, the raw experimental data were first arranged into a decision matrix and normalized using vector normalization. In this method, each value was divided by the square root of the sum of squares of all alternatives for the corresponding criterion, ensuring that all criteria were comparable. Equal weights of 0.25 were then assigned to the four mechanical properties to obtain the weighted normalized matrix. Next, the positive ideal solution (best value for each criterion) and the negative ideal solution (worst value) were determined from the weighted normalized matrix. The Euclidean distances of each composite from both the positive and negative ideal solutions were calculated. Finally, the closeness coefficient (CC) was determined as the ratio of the distance from the negative ideal solution to the total distance from both ideal solutions. The composites were ranked by their CC values, with higher CC values indicating superior overall mechanical performance.

PCA was conducted using Python in Google Colab as an independent statistical validation method. Before analysis, the mechanical-property data were standardized to remove scale effects. PCA transformed the correlated mechanical responses into four uncorrelated principal components (PC1–PC4). The composites were then evaluated based on their principal component scores, particularly along the dominant component, which explained the largest variance in the dataset.

#### 2.4.2 RSM design, ANOVA, and desirability optimization

RSM was implemented using Design-Expert software (Version 12.0, Stat-Ease Inc., Minneapolis, MN, USA), with the experimental matrix generated using a central composite design (CCD). RSM was applied to model and optimize the machining performance of the selected C3 composite. No separate one-factor-at-a-time optimization preceded the RSM analysis. Three continuous factors were considered: cutting speed (*V*_c_), feed rate (*f*), and depth of cut (*t*), while the cutting environment was included as a categorical factor with two levels, dry and MQL. The continuous factors were evaluated at *V*_c_ = 80, 123.5, and 167 m min^−1^; *f* = 0.10, 0.13, and 0.16 mm per rev; and *t* = 1.00, 1.25, and 1.50 mm. The CCD comprised 26 experimental trials, including replicated center points to estimate experimental error and evaluate model adequacy.

Surface roughness (*R*_a_), cutting force (*F*_c_), chip reduction coefficient (*ξ*), and cutting temperature (*T*) were selected as responses representing surface quality, cutting load, chip deformation, and thermal response, respectively. Second-order regression models were developed for each response and evaluated using analysis of variance (ANOVA) at a 95% confidence level. Model adequacy was assessed using lack of fit, *R*^2^, adjusted *R*^2^, predicted *R*^2^, adequate precision, mean absolute percentage error (MAPE), and residual diagnostics. Multi-response optimization was then performed using the desirability-function approach, minimizing *R*_a_, *F*_c_, *ξ*, and *T* and assigning them equal importance.

## 3 Results and discussion

### 3.1 Mechanical characterization

#### 3.1.1 Tensile strength

Fig. 4 shows a clear composition-dependent tensile response for the hybrid Al-MMCs. The unreinforced Al sample C1 exhibited the lowest tensile strength of 69.7 MPa, highlighting the limited resistance of the monolithic Al matrix to plastic deformation and crack initiation. With the addition of hybrid reinforcements, the tensile strength increased to 153.3 MPa for C2 and reached a maximum of 201.5 MPa for C3, corresponding to improvements of about 120% and 189%, respectively, compared with C1. The maximum tensile strength of C3 (6SiC–2Gr–2ZnO) is attributed to the favorable balance among the three reinforcements. At 6 wt% SiC, a sufficient ceramic fraction is available for direct load transfer and matrix constraint and for promoting dislocation accumulation associated with the elastic and thermal-expansion mismatch between SiC and Al.^37,38^ The 2 wt% Gr fraction can further assist stress transfer, impede dislocation motion, and promote crack deflection when adequately dispersed,^39,40^ while 2 wt% ZnO contributes fine-particle strengthening and resistance to localized plastic deformation.^41^ The compact C3 microstructure observed in Section 3.1.5, with no severe clustering or large casting voids at the examined locations, is consistent with efficient operation of these strengthening mechanisms.

**Fig. 4:**
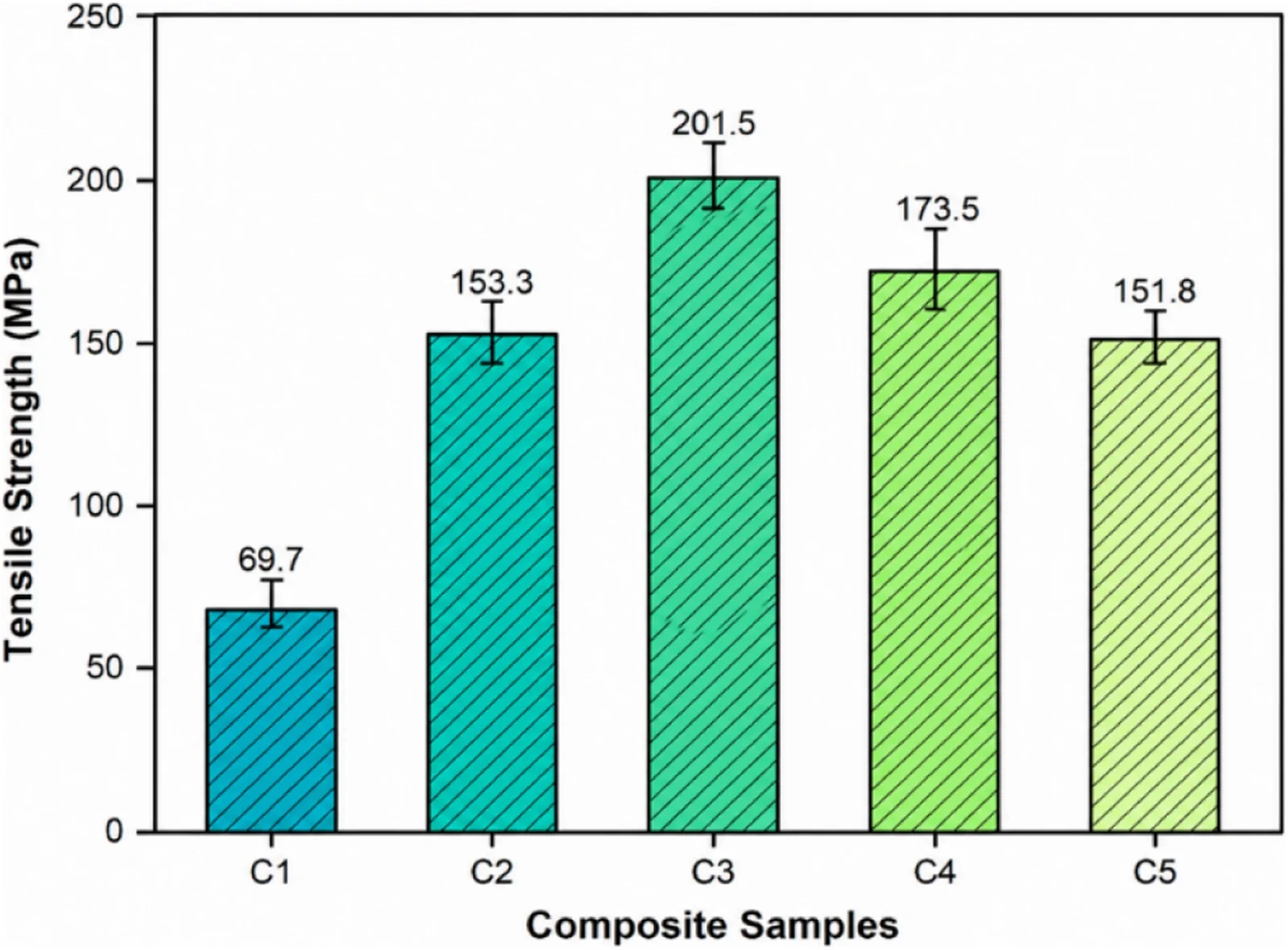
Effect of hybrid reinforcement composition on the tensile strength of fabricated Al-MMCs (C1–C5).

Beyond C3, the tensile strength decreased to 173.5 MPa for C4 and 151.8 MPa for C5, corresponding to reductions of approximately 13.9% and 24.7%, respectively, relative to C3. Because the total reinforcement content remained fixed at 10 wt%, the change from C3 (6SiC–2Gr–2ZnO) to C5 (2SiC–4Gr–4ZnO) reduced the SiC fraction by two-thirds while doubling both Gr and ZnO. The lower SiC content reduces the contribution from the principal rigid load-bearing phase, including direct load transfer, matrix constraint, and thermal-mismatch-induced dislocation strengthening.^37,38^ At the same time, the higher Gr and ZnO fractions increase the total interfacial area requiring effective wetting and interfacial contact with the molten Al matrix. The higher Gr fraction is particularly relevant to the C5 response. Gr platelets are susceptible to restacking and agglomeration because of van der Waals attraction, while their poor wettability by molten Al can hinder infiltration of Gr-rich clusters. Consequently, solidification-processed Al–Gr composites show increased agglomeration and porosity and reduced properties when the Gr fraction exceeds an optimum range.^16,39^ Such defects reduce the effective matrix–reinforcement contact area and provide preferential sites for crack initiation. C5 contains 4 wt% Gr compared with 2 wt% in C3 and is therefore more susceptible to these concentration-dependent effects, although the present study did not directly examine the C5 microstructure.

At the casting temperature of approximately 750–800 °C, carbon at Gr edges and defect sites can react locally with Al to form Al_4_C_3_.^16,39^ A limited interfacial reaction can improve local anchoring and load transfer, whereas extensive or discontinuous Al_4_C_3_ formation may consume Gr and introduce a brittle phase that facilitates crack initiation. The higher Gr content in C5 provides a larger potential Al–Gr reaction area, increasing the likelihood of these adverse effects. Increasing ZnO from 2 wt% in C3 to 4 wt% in C5 can also affect reinforcement efficiency. At higher nanoparticle contents, particle aggregation and porosity can offset the strengthening contribution of ZnO. Fatile *et al.*,^24^ for example, reported maximum tensile strength at 4 wt% ZnO followed by a decrease at 5 wt% and associated the decline with nanoparticle aggregation, grain-boundary saturation, and porosity. In addition, Al has a greater affinity for O_2_ than Zn, making the aluminothermic reaction 2Al + 3ZnO → Al_2_O_3_ + 3Zn thermodynamically feasible.^42^ Its extent in liquid Al depends on temperature, wetting, contact time, and reaction kinetics. If local reaction occurred during casting, nonuniform Al_2_O_3_-rich and Zn-containing regions could further disrupt interfacial continuity.

The lower tensile strength of C5 can therefore be attributed primarily to the substantial reduction in SiC-mediated load bearing combined with the higher Gr and ZnO fractions, which increase susceptibility to particle agglomeration, incomplete wetting, porosity, and less uniform matrix–reinforcement contact. Possible Al–C and Al–ZnO interfacial reactions may additionally promote the formation of brittle or compositionally nonuniform interfacial regions. These effects increase local stress concentration and promote earlier crack initiation, outweighing the additional strengthening expected from increasing Gr and ZnO. C5 nevertheless remains considerably stronger than the unreinforced C1 sample, indicating that reinforcement strengthening remains active, while C3 provides the most favorable reinforcement balance for tensile loading.

The observed trend is consistent with earlier studies on Al-based composites. Wang *et al.*^12^ reported a maximum tensile strength of about 171.3 MPa in Al-GNS (Gr nanosheets)–SiC composites when GNS and SiC were uniformly distributed. Studies on Al–SiC nanocomposites have also identified load transfer, Orowan strengthening, thermal-mismatch strengthening, and increased dislocation density as important strengthening mechanisms.^37,38^ However, Gr–reinforced Al systems are highly sensitive to dispersion because Gr can agglomerate and phase-separate in metallic matrices, reducing its strengthening effect at higher contents.^39^ Overall, the tensile response indicates that C3 provides the most favorable reinforcement balance, whereas further increases in Gr and ZnO, together with reduced SiC content, diminish reinforcement efficiency and lower tensile strength.

#### 3.1.2 Flexural strength

Fig. 5 shows the flexural strength of Al-MMCs as a function of hybrid reinforcement composition. The unreinforced Al sample C1 exhibited the lowest flexural strength of 193 MPa, indicating the limited bending resistance of the monolithic Al matrix. With reinforcement addition, the flexural strength increased to 262.3 MPa for C2 and reached the maximum value of 283.3 MPa for C3, corresponding to improvements of 35.9% and 46.8%, respectively, compared with C1. C3 was also about 8.0% stronger than C2 under flexural loading. The superior flexural response of C3 can be related to the stress distribution developed during three-point bending. One surface of the specimen is subjected to tensile stress while the opposite surface experiences compression, making resistance to matrix deformation, load transfer, and crack initiation important. In C3, the 6 wt% SiC fraction provides a rigid load-bearing contribution and restricts local matrix deformation, while 2 wt% Gr can help redistribute stress and deflect crack paths when adequately dispersed. The 2 wt% ZnO fraction provides additional particle-scale resistance to localized deformation. The compact matrix and absence of severe agglomeration or large casting voids at the examined C3 locations are consistent with effective load sharing among the matrix and reinforcements.^38,43^ However, these observations do not quantify interfacial bond strength or demonstrate superior bonding relative to the other compositions.

**Fig. 5:**
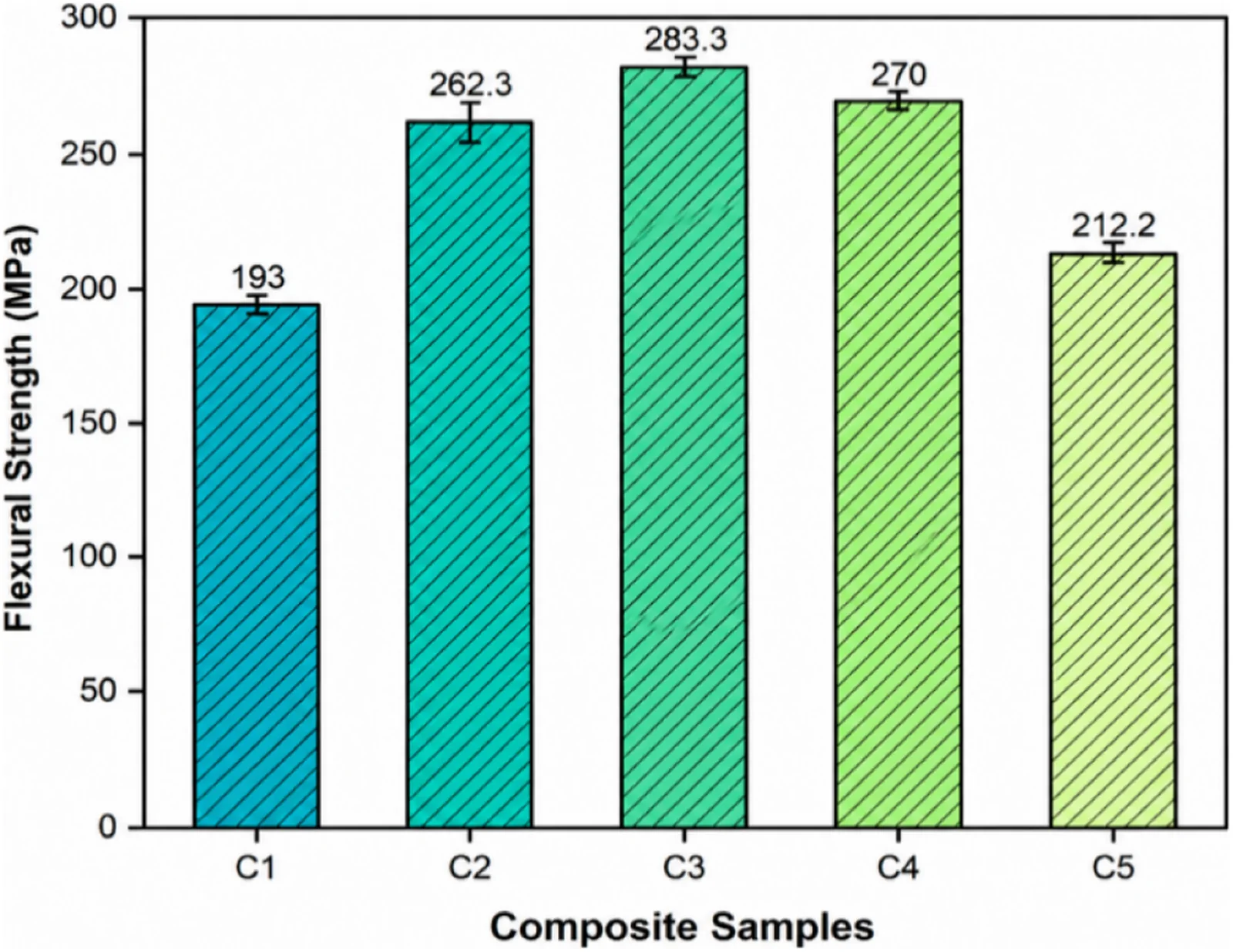
Effect of hybrid reinforcement composition on the flexural strength of fabricated Al-MMCs (C1–C5).

Beyond C3, the flexural strength decreased to 270 MPa for C4 and 211.2 MPa for C5, corresponding to reductions of approximately 4.7% and 25.5%, respectively, relative to C3. Because the total reinforcement content remained fixed at 10 wt%, progression from C3 to C5 simultaneously reduced SiC from 6 to 2 wt% and increased both Gr and ZnO from 2 to 4 wt%. The lower SiC fraction reduces the contribution of the principal rigid load-bearing phase and its ability to constrain matrix deformation. At the same time, higher Gr and ZnO fractions increase the interfacial area requiring effective wetting and dispersion. Higher Gr contents can increase susceptibility to restacking and agglomeration,^39^ while elevated nanoparticle contents can promote particle aggregation and porosity.^24^ Under flexural loading, such discontinuities are particularly detrimental in the tensile region because they act as local stress concentrators and facilitate earlier crack initiation. These concentration-dependent effects plausibly explain the larger reduction in C5. This trend is consistent with previous studies; for example, Rumi and Rahman^10^ reported a 52.5% reduction in flexural strength after ZnO addition to an Al–Al_2_O_3_ composite and associated the decrease with a more brittle fracture response. Papabathina *et al.*^44^ similarly reported the highest flexural strength at 6 wt% hybrid reinforcement in an Al6061/SiC/graphite system, indicating that increasing reinforcement content does not necessarily produce a continuous improvement in bending strength. Overall, the flexural response indicates that C3 provides the most favorable reinforcement balance for bending resistance, whereas further increases in Gr and ZnO, along with reduced SiC content, lower load-transfer efficiency and increase susceptibility to defect-assisted crack initiation.

#### 3.1.3 Impact strength

Fig. 6 shows that the impact strength of Al-MMCs varied with hybrid reinforcement composition. The unreinforced Al sample C1 exhibited the lowest impact strength of 31.5 kJ m^−2^, indicating limited resistance to sudden impact loading and dynamic crack growth. With reinforcement addition, the impact strength increased to 37 kJ m^−2^ for C2 and reached the maximum value of 43.3 kJ m^−2^ for C3, corresponding to improvements of about 17.5% and 37.5%, respectively, compared with C1. The superior impact response of C3 is attributed to the balanced combination of 6 wt% SiC, 2 wt% Gr, and 2 wt% ZnO, which improved stress distribution, delayed crack initiation, and increased the energy required for fracture. The improvement in C3 is consistent with SiC's strengthening role as a hard, load-bearing phase that restricts localized plastic deformation when well bonded to the Al matrix.^38,43,45^ Gr can further assist energy absorption by supporting stress transfer and hindering dislocation motion when uniformly dispersed, while ZnO can provide additional particle strengthening and local resistance to deformation.^39,40,46^ This explanation agrees with the SEM observations in Section 3.1.5, where C3 showed a compact matrix, embedded reinforcement-rich regions, no severe particle clustering, no large casting voids, and no clear interfacial gaps. Such features support effective matrix–reinforcement bonding and a more stable crack-propagation path under impact loading.^43,45^ Similar studies on Al/SiC nanocomposites also show that homogeneous reinforcement dispersion and strong interfacial bonding are critical for improving mechanical performance.^47^

**Fig. 6:**
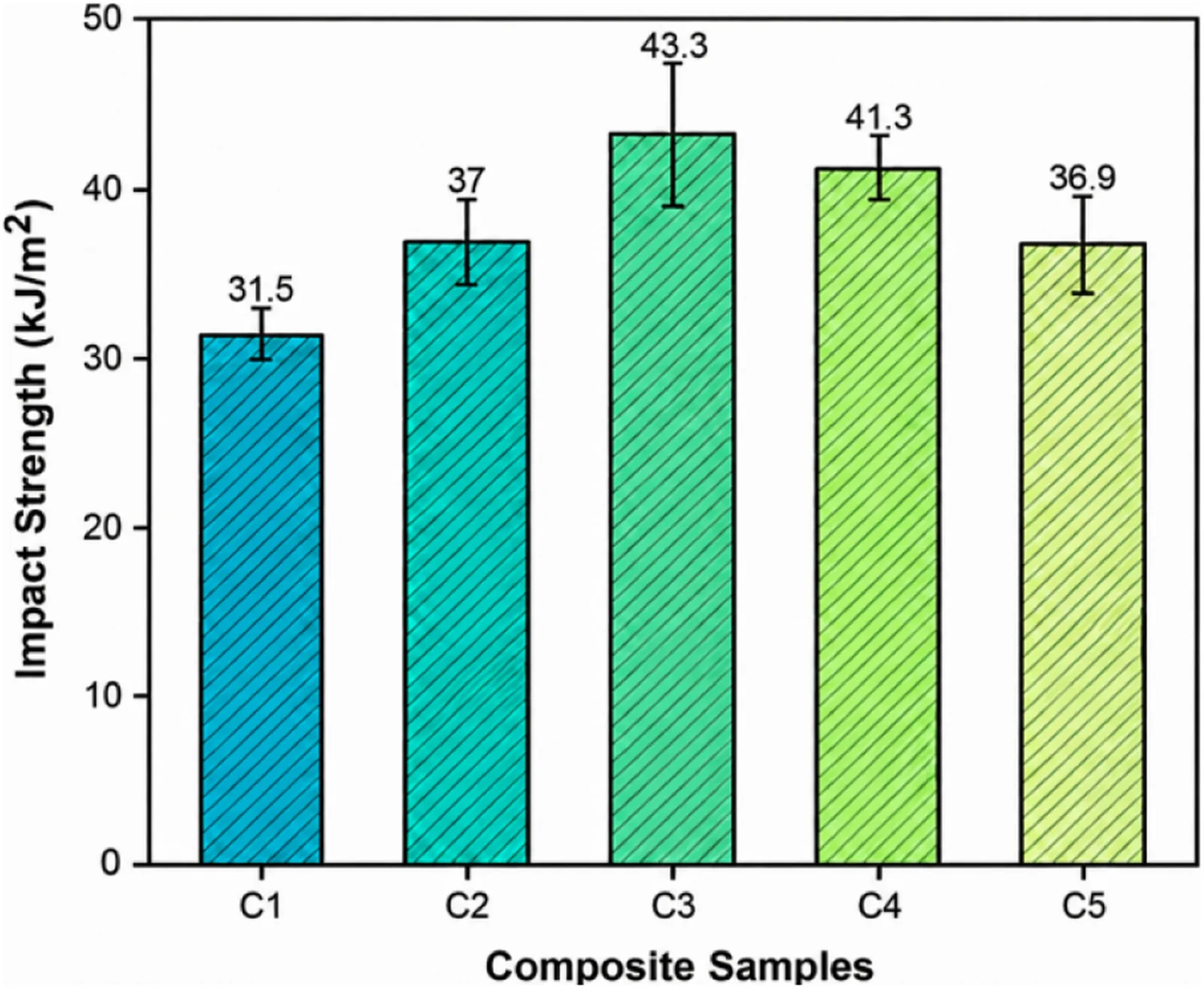
Effect of hybrid reinforcement composition on the impact strength of fabricated Al-MMCs (C1–C5).

Beyond the optimum C3 composition, the impact strength decreased slightly to 41.3 kJ m^−2^ for C4 and then to 36.9 kJ m^−2^ for C5. Although C4 still retained higher impact strength than C1 and C2, its reduction relative to C3 suggests that increasing Gr and ZnO beyond 2 wt% each, together with reducing SiC from 6 to 4 wt%, weakened the most effective load-bearing and crack-resisting balance. The further decline in C5 is attributed to the combined effect of lower SiC content and higher nano-reinforcement content, which can increase particle clustering, porosity, and local interfacial weakness. These defects act as stress concentrators, reduce the effective energy-absorbing area, and promote premature crack initiation during impact loading.^43,45,48,49^ This trend agrees with a study,^10^ which found that adding ZnO to an Al–Al_2_O_3_ composite reduced impact strength by 12%, attributed to a more brittle fracture marked by cleavage cracks, deep shear dimples, and crystallographic planes. Conversely, the beneficial effect of a controlled SiC/Gr hybrid system is supported by Lakshmaiya *et al.*,^50^ who reported an impact strength of about 32 kJ m^−2^ and an 88% improvement over the base alloy for an Al–SiC/Gr composite. The present C3 value of 43.3 kJ m^−2^ is higher, which may be associated with the additional contribution of ZnO and the more balanced SiC–Gr–ZnO ratio. Overall, C3 exhibited the highest impact strength among the investigated compositions. This superior performance arises from the combined effects of reinforcement proportion, dispersion quality, interfacial integrity, and energy-dissipation processes.

#### 3.1.4 Hardness

Fig. 7 shows the hardness variation of Al-MMCs with hybrid reinforcement composition. The unreinforced Al sample C1 exhibited the lowest hardness of 29.2 HV, highlighting the limited resistance of the soft Al matrix to localized plastic deformation under indentation. With reinforcement, hardness increased sharply to 71.7 HV for C2, an improvement of about 145.5% over C1. This highest value is mainly attributed to the 8 wt% SiC content in C2, since SiC is a hard ceramic phase that improves indentation resistance by restricting localized matrix deformation and increasing resistance to dislocation motion.^38,43,47^ The reinforcement synergism observed for tensile, flexural, and impact performance does not imply that the same composition will maximize hardness. Hardness is governed primarily by resistance to localized indentation and is therefore particularly sensitive to the fraction of the hard SiC phase. C2 contains the highest SiC content (8 wt%) and consequently exhibits the maximum hardness of 71.7 HV. As SiC decreases to 6, 4, and 2 wt% in C3, C4, and C5, respectively, hardness decreases to 69.3, 67.7, and 55.3 HV, corresponding to reductions of approximately 3.3%, 5.6%, and 22.9% relative to C2. This trend indicates that the increasing Gr and ZnO fractions do not fully compensate for the progressive loss of SiC-mediated indentation resistance.

**Fig. 7:**
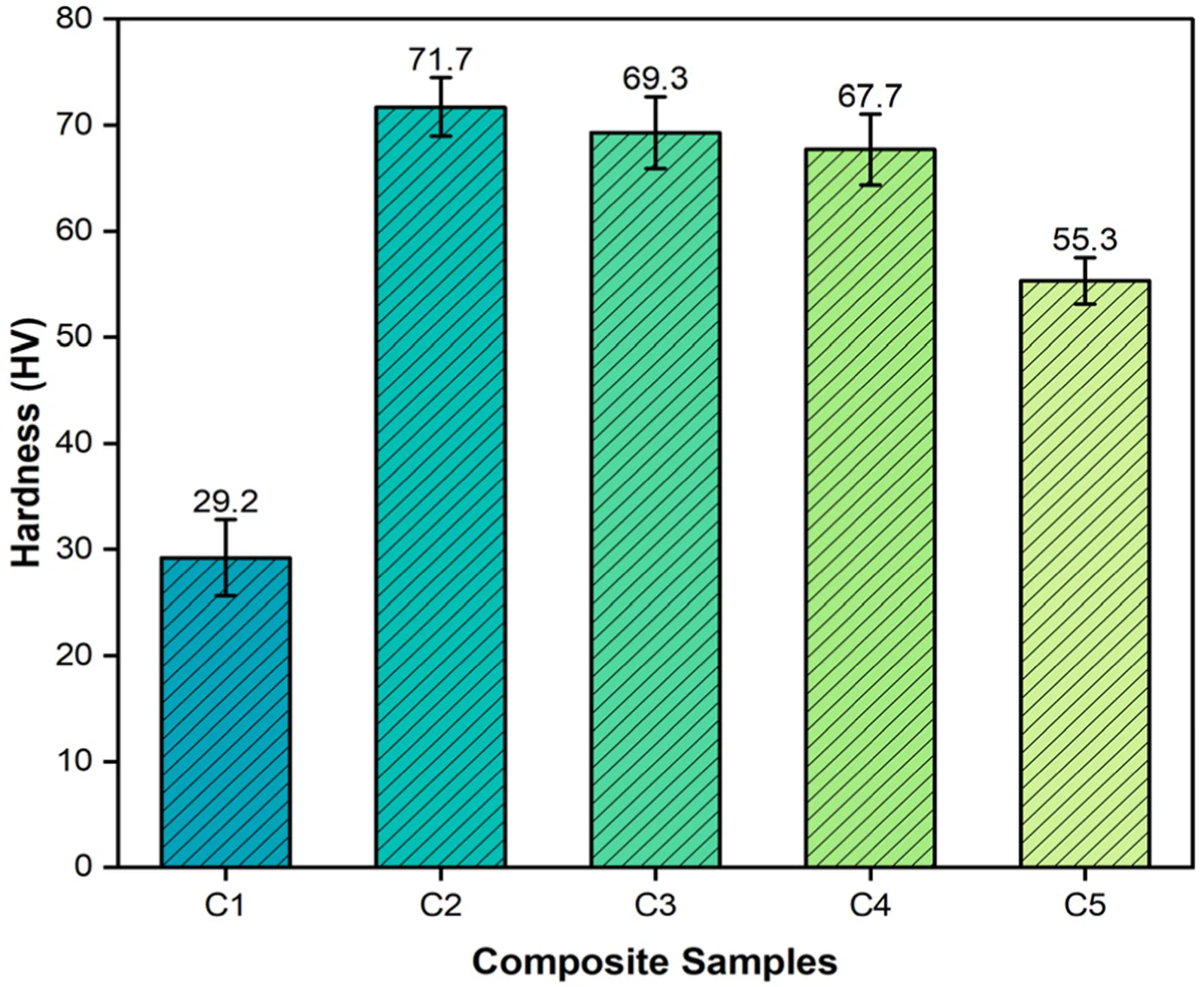
Effect of hybrid reinforcement composition on the hardness of the fabricated Al-MMCs (C1–C5).

The hardness improvement in the reinforced composites can be explained by the combined effects of hard-particle strengthening, restricted plastic flow, dislocation obstruction, and matrix–reinforcement load transfer.^38,43,45,47^ SiC provided the dominant indentation-resistance effect, while ZnO contributed additional oxide-particle strengthening when adequately dispersed.^41^ Gr may also assist strengthening through stress transfer and dislocation restriction at controlled contents, although excessive Gr can agglomerate and reduce strengthening efficiency.^39,40,46^ This mechanism agrees with the SEM observations in Section 3.1.5, where C3 showed embedded reinforcement-rich regions, no severe particle clustering, no large casting voids, and no clear interfacial gaps. Such features support effective matrix–particle bonding, which explains why C3 maintained high hardness while exhibiting superior tensile, flexural, and impact performance.

The decline in hardness from C3 to C5 is attributed to the reduced SiC fraction and the increasing tendency for Gr/ZnO agglomeration, porosity formation, and local interfacial weakness at higher nano-reinforcement contents. These defects disrupt Al matrix continuity, reduce effective particle–matrix contact, and facilitate localized deformation during indentation.^43,45^ In addition, higher Gr fractions can increase susceptibility to agglomeration and reduce effective particle–matrix contact, facilitating localized deformation during indentation and contributing to the lower hardness of C5.^39,46^ Similar hardness improvement has been reported for AA6061/SiC/Gr hybrid composites,^50^ while Bhoi *et al.*^41^ found that Al–ZnO nanorod composites showed improved hardness and nanohardness when ZnO was distributed with a clear interface. These findings confirm that SiC content predominantly controls hardness, while ZnO and Gr are beneficial only when their contents remain low enough to avoid defect-assisted softening. Overall, SiC content predominantly governs indentation resistance, while Gr and ZnO provide complementary strengthening at controlled fractions. C2 therefore exhibits the maximum hardness, whereas C3 offers the best balance between hardness and the other measured mechanical properties.

#### 3.1.5 SEM and fractographic analysis

The C3 composite (90 wt% Al, 6 wt% SiC, 2 wt% ZnO, and 2 wt% Gr) was examined under SEM because it showed the best overall mechanical performance. Fig. 8a and b show a compact Al matrix without large casting voids, severe particle clustering, or clear interfacial gaps; the parallel traces are likely polishing marks. The bright angular regions are consistent with ceramic particles, whereas dark regions may indicate small pores or matrix–reinforcement contrast. The embedded reinforcement-rich regions suggest good interfacial integrity and load transfer, consistent with reports that Al/SiC composite properties depend significantly on SiC morphology, particle distribution, and Al–SiC bonding, while reinforcement–matrix interfaces govern strengthening and damage resistance in Al-MMCs.^43,45^ The compact matrix and lack of severe agglomeration or large voids at the examined C3 locations also align with conditions favorable for the strengthening mechanisms discussed in Sections 3.1.1–3.1.4 and with the balanced mechanical response of C3. However, the FESEM observations do not quantify interfacial bond strength or demonstrate improved bonding relative to C2, C4, or C5. Furthermore, because only selected 2D regions were examined, and no compositional mapping or 3D imaging was performed, the images do not demonstrate bulk-scale homogeneity or phase-specific reinforcement distribution throughout the composite.

**Fig. 8:**
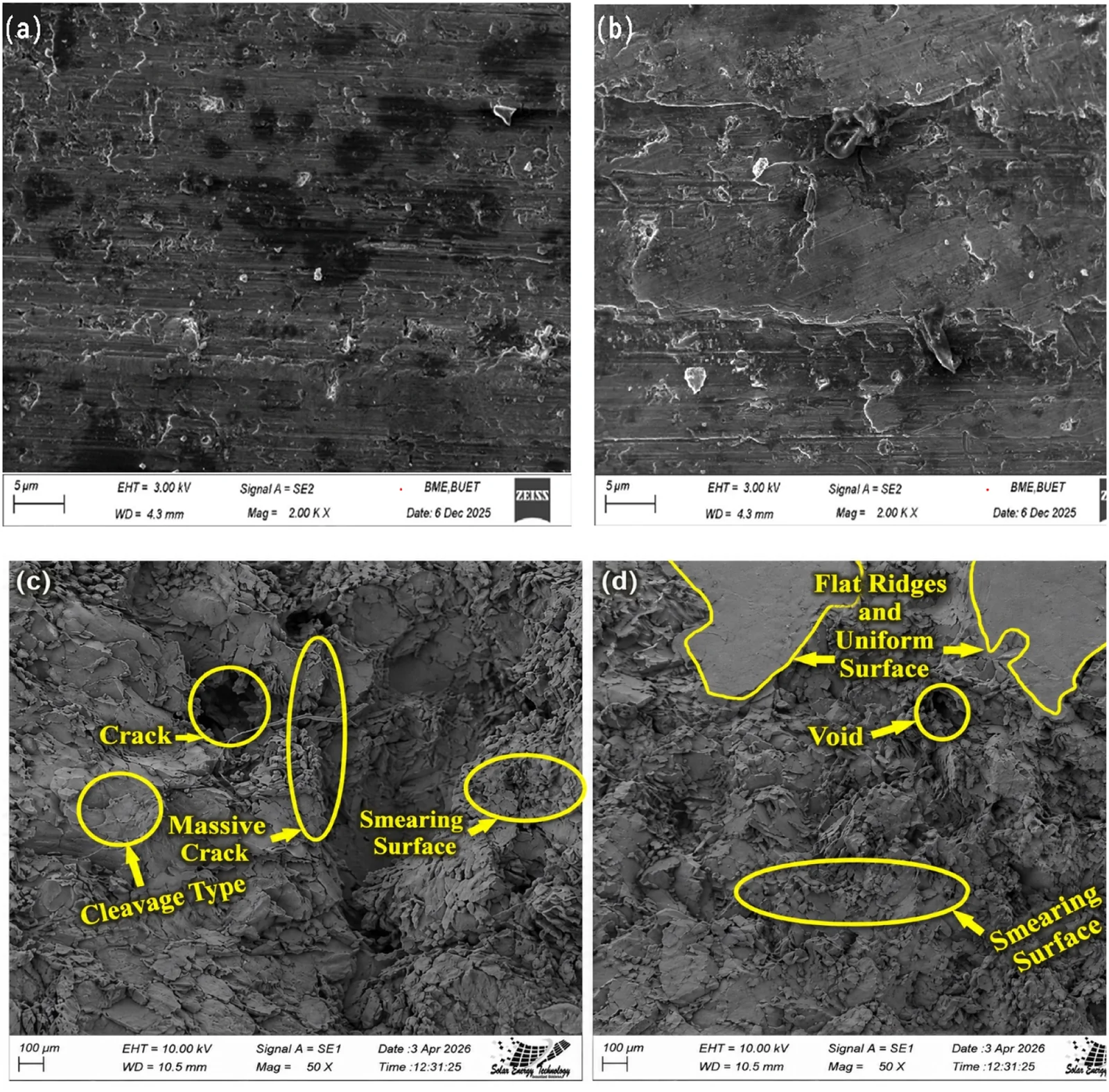
SEM micrographs and fracture-surface morphology of the selected C3 hybrid Al-MMC: (a) low-magnification surface morphology, (b) high-magnification reinforcement-rich region, (c) fracture surface showing major crack, secondary cracks, cleavage-like regions, and smeared matrix surface, and (d) fracture surface showing flat ridges, void formation, and localized matrix deformation.

Fig. 8c and d indicate a mixed ductile and quasi-cleavage fracture mode in C3. The major cracks, secondary cracks, cleavage-like facets, and smeared matrix regions in Fig. 8(c) suggest crack initiation at local stress concentrators, mainly hard particles, pores, or weak particle–matrix interfaces. This behavior is typical of particle-reinforced Al-MMCs, where elastic–plastic mismatch promotes interface stress concentration, particle cracking, and interfacial debonding.^48,49^Fig. 8(d) supports this mechanism: planar ridges indicate localized quasi-cleavage, whereas voids and smeared Al-rich regions indicate microvoid nucleation, growth, and coalescence. Thus, C3 did not fail by a purely brittle route; the Al matrix retained local plastic deformation before rupture. Similar mixed fracture involving dimple rupture, shear failure, interface cracking, and quasi-cleavage has been reported for hybrid Al6061 composites.^51^ Overall, the C3 response is consistent with balanced reinforcement distribution and adequate interfacial integrity, with fracture progressing through combined matrix deformation, particle/interface cracking, and quasi-cleavage propagation.

### 3.2 Overall mechanical performance ranking

#### 3.2.1 TOPSIS-based ranking

Table 3 presents the TOPSIS ranking of the fabricated Al-MMCs based on tensile strength, flexural strength, impact strength, and hardness as the beneficial criteria. C3 achieved the highest CC (0.967) and was ranked first, followed by C4, C2, C5, and C1. This result confirms that C3 is the most balanced composite rather than the best in only one property. Its small distance from the positive ideal solution (0.004) and large distance from the negative ideal solution (0.131) indicate that C3 is closest to the best combined mechanical response among the evaluated alternatives. This agrees with the TOPSIS principle, where the preferred option should be closest to the positive ideal solution and farthest from the negative ideal solution.^52,53^

**Table 3:** TOPSIS analysis of fabricated composites

Composites	Weighted tensile strength	Weighted flexural strength	Weighted impact strength	Weighted hardness	Distance from ideal solution (+)	Distance from ideal solution (−)	Closeness coefficient	Rank
C1	0.050	0.088	0.092	0.054	0.134	0.000	0.000	5
C2	0.110	0.119	0.108	0.132	0.040	0.105	0.722	3
**C3**	**0.144**	**0.128**	**0.127**	**0.128**	**0.004**	**0.131**	**0.967**	**1**
C4	0.124	0.122	0.121	0.125	0.023	0.112	0.830	2
C5	0.109	0.096	0.108	0.102	0.060	0.078	0.565	4

The ranking also explains why C2, despite showing the highest hardness, did not emerge as the optimum composition. C2 obtained a high weighted hardness value (0.132), but its weighted tensile, flexural, and impact values were lower than those of C3. In contrast, C3 provided the highest weighted tensile, flexural, and impact responses, together with only a slight hardness reduction relative to C2. Thus, the TOPSIS outcome indicates that C3 offers a better compromise among strength, toughness, and hardness. C4 ranked second because its mechanical values remained close to those of C3, but reductions in tensile, flexural, and impact strengths, and in hardness increased its distance from the ideal solution. C5 and C1 ranked lower because they were farther from the positive ideal solution, mainly due to reduced reinforcement efficiency in C5 and the absence of reinforcement in C1. Similar TOPSIS-based studies on composite materials have used tensile, flexural, impact, and hardness responses as beneficial attributes to identify the best overall composition rather than relying on a single mechanical property.^54,55^

#### 3.2.2 Principal component analysis

Table 4 presents the PCA scores for the fabricated composites, and Fig. 9 provides a visual comparison in a radar chart. PC1 accounted for 92.13% of the total variance, indicating that a single dominant component captured most of the variation in mechanical performance. PC2, PC3, and PC4 contributed only 4.61%, 3.14%, and 0.11%, respectively. Therefore, PC1 can be considered the main descriptor of the combined mechanical performance. This aligns with PCA, which transforms correlated variables into orthogonal components and captures most dataset variation in the first few components.^56^ Thus, PC1-based ranking effectively represents the dominant performance trend.

**Table 4:** PCA scores, variance, and ranking of fabricated composites

Composites	PC1	PC2	PC3	PC4	Rank
C1	−3.364	−0.059	0.333	0.011	5
C2	0.583	0.818	−0.106	0.030	3
C3	2.133	−0.381	0.233	0.084	1
C4	1.327	−0.059	0.186	−0.121	2
C5	−0.679	−0.319	−0.646	−0.004	4
Variance (%)	92.13	4.61	3.14	0.11	—

**Fig. 9:**
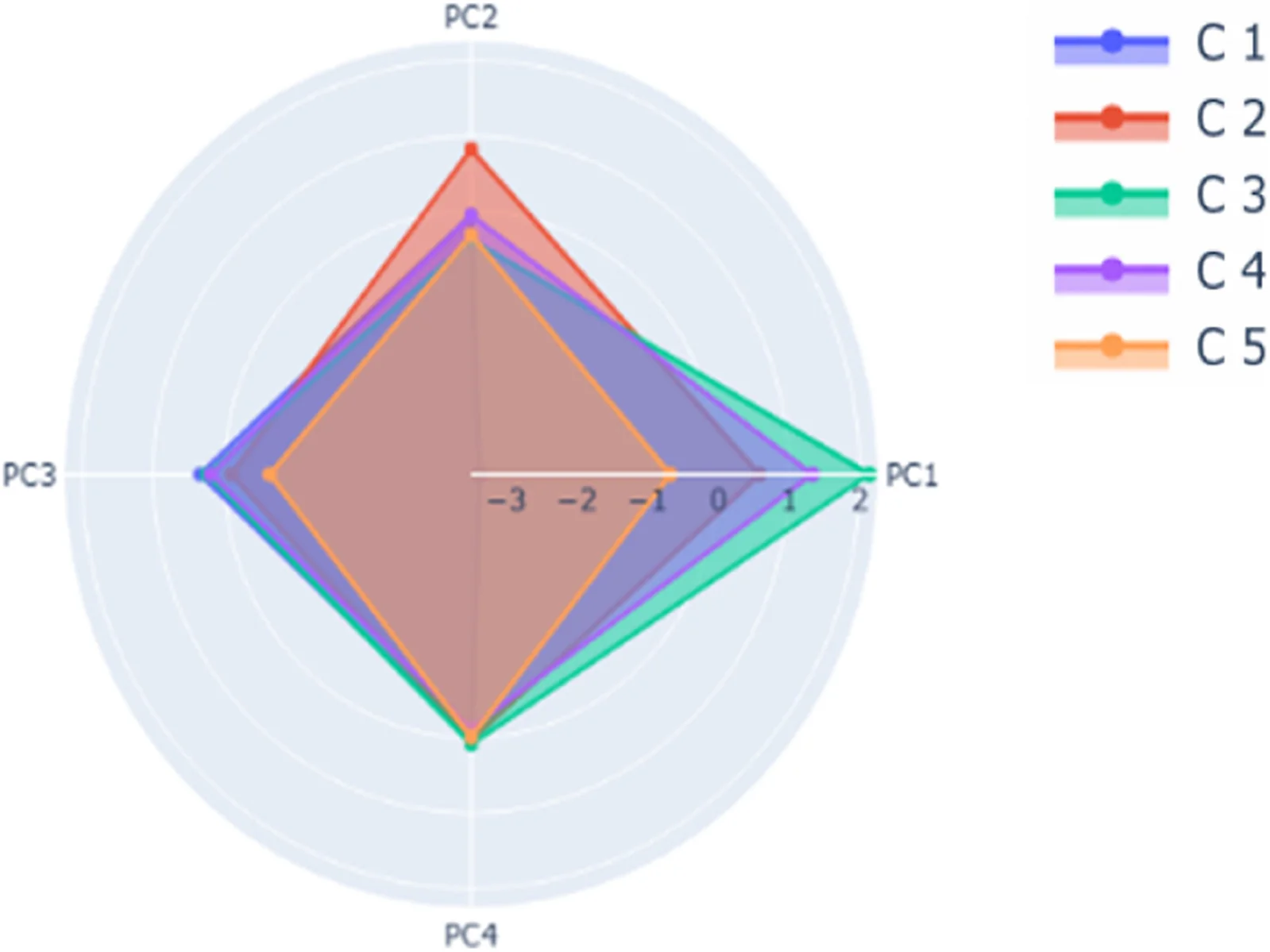
Radar chart of PCA scores for the fabricated composites.

Based on PC1 scores, C3 ranked first with the highest value of 2.133, followed by C4, C2, C5, and C1. This ranking matches the TOPSIS result, confirming the robustness of the selection. The high PC1 score of C3 is linked to its superior tensile, flexural, and impact strengths, as well as its high hardness. Although C2 showed the highest hardness, its lower tensile, flexural, and impact strengths limited its PC1 score to 0.583, placing it third. This difference is important because hardness alone does not fully reflect the mechanical performance required for structural applications. C4 ranked second because it retained relatively high values across all four properties, but it did not exceed C3 in any strength or impact response. C5 and C1 showed negative PC1 scores, indicating weaker overall mechanical performance compared with the reinforced compositions C2–C4.

Fig. 9 further supports this ranking. The radar chart shows that C3 extends farthest along PC1, the most influential component. The smaller contributions of PC2, PC3, and PC4 indicate that secondary variations among the composites had a limited influence on the final ranking. Therefore, the PCA results confirm that the optimal composition is not determined solely by maximum hardness, but by a balanced combination of tensile strength, flexural strength, impact resistance, and indentation resistance. The agreement between TOPSIS and PCA supports selecting C3 for subsequent machining assessment, as both independent methods produced the same ranking order: one based on distance from ideal solutions and the other on variance-driven statistical projection.

### 3.3 Machining performance assessment

The machinability of the selected C3 composite, containing 90 wt% Al, 2 wt% Gr, 2 wt% ZnO, and 6 wt% SiC, was evaluated under dry and MQL-assisted turning to understand the effect of lubrication on surface quality, cutting force, chip deformation, and thermal response. Fig. 10 compares the variations in *R*_a_, *F*_c_, *ξ*, and *T* with *V*_c_, *f*, and *t*. The results show that MQL consistently improves machining performance compared with dry cutting, mainly by reducing friction, adhesion, built-up edge formation, chip-tool contact severity, and heat generation in the cutting zone. This behavior is consistent with prior studies on MQL-assisted turning of Al-MMCs and hybrid Al composites, where improved lubrication at the tool–chip and tool–workpiece interfaces reduced cutting force, surface roughness, tool wear, and cutting temperature.^19,21,22,57–59^

**Fig. 10:**
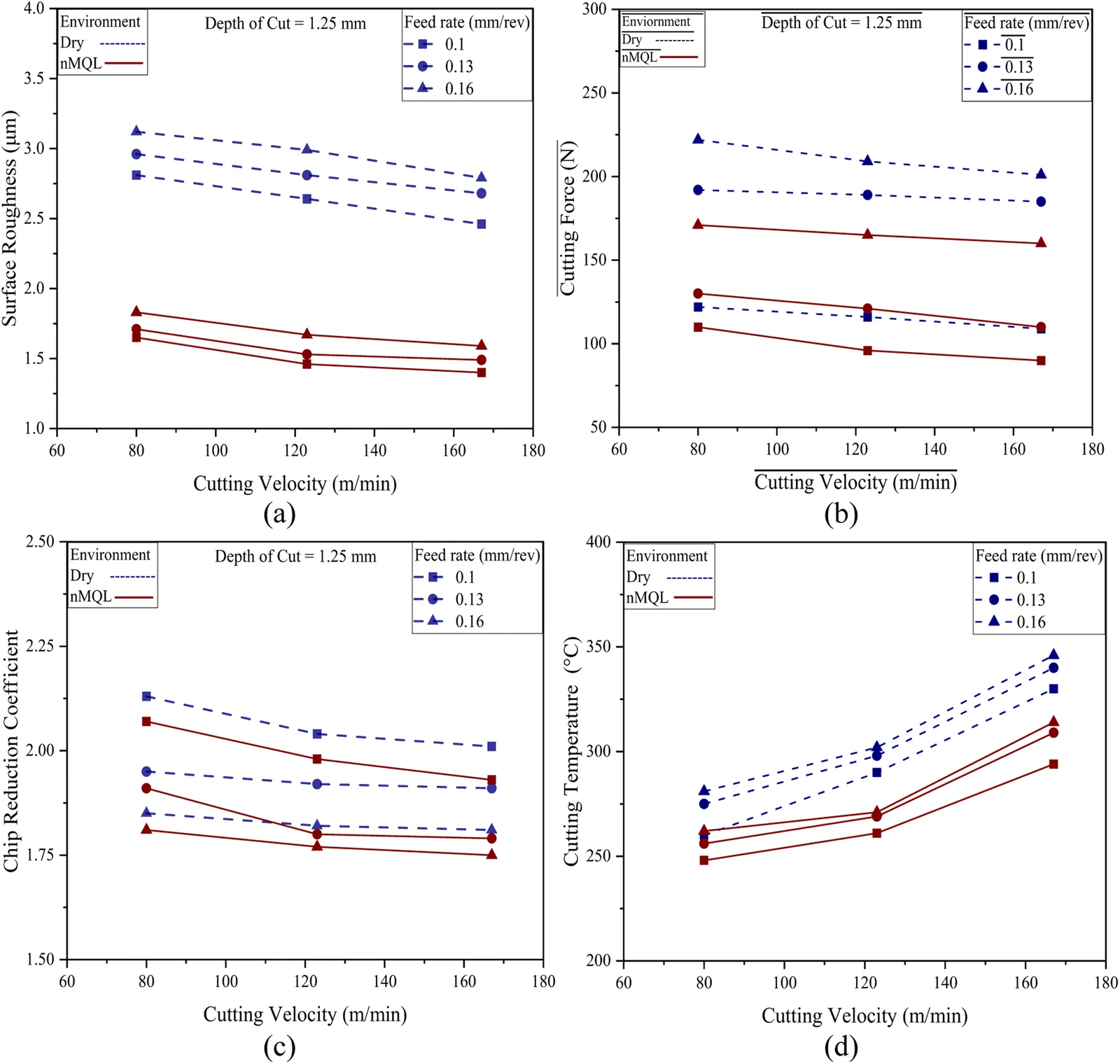
Effect of cutting speed (*V*_c_), feed rate (*f*), and depth of cut (*t*) on the machining responses during turning of the selected Al-MMC: (a) surface roughness, (b) cutting force, (c) chip reduction coefficient, and (d) cutting temperature.

#### 3.3.1 Surface roughness

Fig. 10(a) shows that the surface roughness (*R*_a_) decreases with increasing cutting speed and increases with feed rate under both dry and MQL conditions. Under dry turning, *R*_a_ decreases from 2.81–3.12 µm at 80 m min^−1^ to 2.46–2.79 µm at 167 m min^−1^. Under MQL, the corresponding reduction is from 1.65–1.83 µm to 1.40–1.59 µm. The improvement at higher cutting speeds is attributed to more stable shearing, lower tendency toward built-up edge, reduced chip adhesion, and shorter tool–workpiece contact time. In contrast, increasing the feed rate increases *R*_a_ because a larger uncut chip thickness produces deeper feed marks, greater plowing, and a higher cutting load. MQL reduces *R*_a_ by 41.28–45.55%, with an average reduction of 43.31% compared with dry cutting. This confirms that MQL mist effectively reaches the tool–chip/workpiece interfaces and reduces frictional sliding, local adhesion, and thermal softening of the machined surface. The result agrees with Kannan *et al.*,^22^ who reported improved machinability of Al matrix nanocomposites under MQL, and with Ador *et al.*,^57^ who reported a 24.89% average reduction in *R*_a_ for MQL turning of nanoparticle-reinforced hybrid Al-MMCs. However, Khandey *et al.*^21^ reported that dry cutting was more favorable for surface finish in their Al-MMC system, while MQL was more effective at reducing cutting force. This discrepancy is reasonable because surface formation in Al-MMC turning is strongly affected by reinforcement type, feed range, tool geometry, lubricant access, and the balance between cooling and lubrication. In the present Gr-containing C3 composite, the self-lubricating effect of Gr and the external MQL lubrication likely acted together to suppress adhesion and built-up edge, resulting in consistently lower *R*_a_ under MQL.

#### 3.3.2 Cutting force

The cutting force (*F*_c_) response in Fig. 10(b) decreases with increasing cutting speed and increases with feed rate for both cutting environments. Under dry cutting, *F*_c_ ranges from 109 to 222 N, whereas under MQL it decreases to 90–171 N. The reduction in *F*_c_ at higher cutting speed can be linked to easier chip flow, lower apparent shear strength in the cutting zone, and reduced contact time. In contrast, a higher feed rate increases *F*_c_ because a larger uncut chip cross-section is sheared, thereby increasing the tool–chip contact length and the normal load on the rake face. MQL lowers *F*_c_ by 9.84–40.54%, with an average reduction of 24.19% relative to dry turning. The reduction is mainly due to lower tool–chip friction, reduced adhesion of the Al matrix to the tool, and improved chip sliding. This trend aligns with Khandey *et al.*,^21^ who reported that MQL reduced *F*_c_ during Al-MMC turning due to its combined cooling and lubrication effects. It also agrees with Kannan *et al.*,^19^ who found that properly optimized cutting speed, feed, and depth of cut reduced force and tool wear during the turning of Al7075/SiC/Gr composites. The present average force reduction is higher than that reported by Ador *et al.*^57^ for a CNT–SiC–Al_2_O_3_ reinforced Al-MMC, where MQL reduced cutting force by 19.63%. This difference may arise from the presence of Gr in the present C3 composite, which can lower interfacial friction, as well as differences in the tested cutting-speed and feed-rate combinations.

#### 3.3.3 Chip reduction coefficient

The chip reduction coefficient (*ξ*) represents chip thickening caused by plastic compression, shear deformation, and friction at the tool–chip interface. As shown in Fig. 10(c), *ξ* decreases with increasing cutting speed under both dry and MQL conditions, indicating lower chip compression and more favorable shear deformation at higher speeds. Increasing feed rate also tends to reduce *ξ* within the tested range, which may be associated with a higher uncut chip thickness and a lower relative degree of chip compression. MQL produces lower *ξ* than dry turning across all tested parameter combinations, with reductions of 2.05–6.28% and an average reduction of 3.62%. This confirms that MQL decreases sticking and sliding resistance on the rake face, improves chip flow, and reduces the severity of chip-tool contact. The result agrees closely with that of Ador *et al.*,^57^ who reported a 4.31% average reduction in *ξ* under MQL compared with dry cutting for nanoparticle-reinforced Al-MMCs. The smaller reduction in *ξ* than in *R*_a_ and *F*_c_ is expected because *ξ* is governed not only by lubrication but also by bulk plastic deformation, reinforcement-induced chip segmentation, and cutting geometry. Therefore, MQL improves chip flow, but the hybrid reinforcement system still controls the fundamental chip deformation response.

#### 3.3.4 Cutting temperature

Fig. 10(d) shows that cutting temperature increases with cutting speed and feed rate under both dry and MQL conditions. At 167 m min^−1^, the dry cutting temperature increases from 330 °C at *f* = 0.10 mm per rev to 340 °C at *f* = 0.13 mm per rev and 346 °C at *f* = 0.16 mm per rev. Under MQL, the corresponding values are lower, increasing from 294 °C to 309 °C and 314 °C. The temperature rise with increasing cutting speed is caused by higher plastic deformation rates in the primary shear zone and greater frictional heat generation at the tool–chip interface. Similarly, higher feed rate increases uncut chip thickness, cutting load, chip-tool contact pressure, and frictional sliding, increasing heat generation. Despite these increases, MQL reduces temperature across all tested conditions, yielding 4.62–10.91% reductions and an average reduction of 8.62% compared with dry machining. This reduction is attributed to the targeted delivery of the lubricant mist into the cutting zone, which lowers frictional heat generation and improves local heat removal. A study^57^ reported an average 9.36% reduction in cutting temperature during MQL-assisted machining of nanoparticle-reinforced Al-MMCs. This reduction is also consistent with MQL review studies showing that MQL can improve surface quality, reduce tool wear, lower cutting force, and control cutting temperature while using much less fluid volume than conventional flood cooling.^58,59^

#### 3.3.5 Tool wear

Tool wear was evaluated using average flank wear, *V*_B_, under the severe cutting condition of *t* = 1.5 mm, *f* = 0.16 mm per rev, and approximately 1200 rpm. As shown in Fig. 11(a), *V*_B_ increased continuously with machining time under both dry and MQL conditions, confirming progressive degradation of the cutting edge during turning of the C3 hybrid Al-MMC. However, dry machining produced a substantially higher wear rate than MQL. Under dry cutting, *V*_B_ increased from 0.041 mm at 1 min to 0.31 mm at 30 min, whereas under MQL, it increased only from 0.024 mm to 0.21 mm over the same duration. Thus, MQL reduced flank wear by about 41.5% at the initial stage and by 32.3% after 30 min. The difference became even more evident at longer cutting times, where dry cutting reached 0.27 mm at 28 min compared with 0.196 mm under MQL, a 27.4% reduction.

**Fig. 11:**
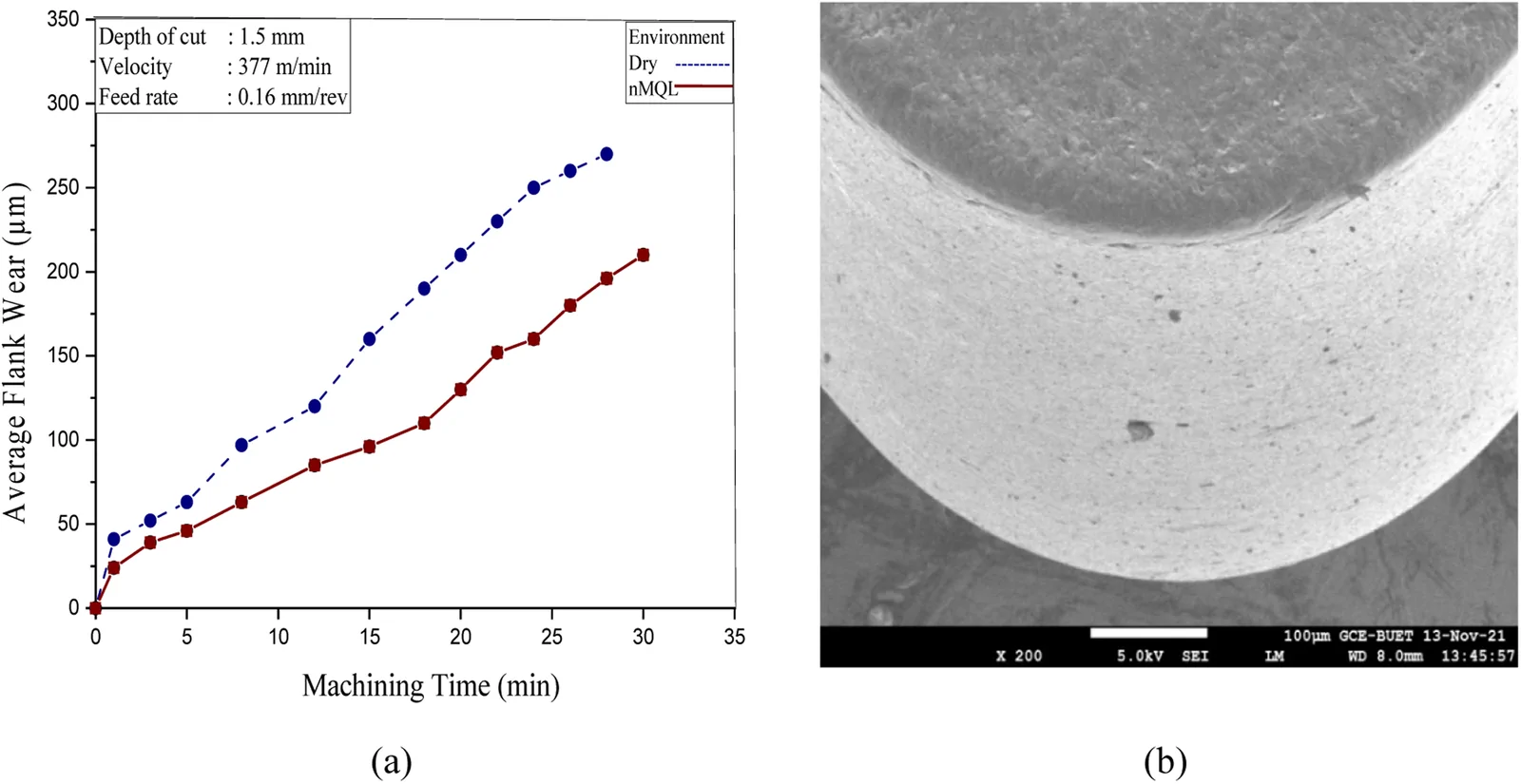
Tool-wear behavior during turning of the selected C3 Al-MMC under dry and MQL conditions: (a) average flank-wear progression with machining time and (b) SEM morphology of the cutting tool after MQL-assisted turning.

The higher wear rate in dry cutting is mainly attributed to the combined action of hard SiC/ZnO particles and adhesive interaction between the Al matrix and the carbide insert. During dry turning, abrasive particles can slide, roll, or become trapped between the tool flank and freshly machined surface, causing micro-grooving and edge deterioration. At the same time, Al adhesion and built-up edge formation can promote unstable chip flow, local tearing, and intermittent removal of adhered material from the tool surface. These mechanisms are widely reported in the machining of particle-reinforced Al-MMCs, where two-body and three-body abrasion, adhesion, and thermally assisted edge damage are dominant contributors to tool wear.^6,20,60^ Recent reviews also confirm that abrasive reinforcement particles in MMCs accelerate tool wear and surface defects during conventional machining.^18,61^

The lower *V*_B_ under MQL can be explained by the ability of the atomized lubricant to reach the tool–chip and tool–workpiece interfaces, forming a thin lubricating film that reduces friction, adhesion, interface temperature, and abrasive contact severity. This mechanism agrees with the reductions in cutting force and temperature reported in Sections 3.3.2 and 3.3.4, respectively, since lower frictional loading and thermal softening directly delay flank wear progression. The SEM image in Fig. 11(b) supports this finding by showing comparatively limited abrasion and moderate edge damage under MQL, rather than severe flank degradation. Similar improvements have been reported by Kannan *et al.*^19^ during turning of Al7075/SiC/Gr hybrid MMCs, where optimized cutting parameters reduced surface roughness, tool wear, and cutting force. Khandey *et al.*^21^ also showed that MQL improves Al-MMC machinability by reducing cutting load and improving tribological conditions. In addition, several studies^19,21,57^ reported lower cutting temperatures, surface roughness, cutting forces, and chip reduction coefficients under MQL for nanoparticle-reinforced hybrid Al-MMCs, supporting the present trend toward wear reduction.

Although MQL substantially reduced wear, the final *V*_B_ of 0.21 mm after 30 min indicates that tool wear was not fully suppressed. This is expected because the C3 composite contains 6 wt% SiC and 2 wt% ZnO, both of which contribute to abrasive interaction during cutting. Therefore, the observed improvement should be understood as reduced wear severity rather than complete prevention of tool degradation. This finding is consistent with studies on Al/SiC_p_ composites, which reported that MQL reduced boundary wear, tool breakage, and major flank wear compared with dry cutting. However, hard ceramic particles remained the main cause of progressive tool damage.^62,63^ Overall, these findings confirm that MQL is an effective cooling-lubrication strategy for reducing flank-wear progression during machining of the Gr–ZnO–SiC–reinforced Al-MMC, while residual wear highlights the need to further optimize the tool material, coating, cutting-edge geometry, and MQL delivery parameters.

#### 3.3.6 Comparative study with literature

Table 5 compares the present Gr–ZnO–SiC–reinforced Al-MMC with Al-based and Zn-based hybrid composites reported in the recent literature. The comparison shows that the present C3 composite offers a balanced combination of mechanical performance and machinability, rather than maximizing a single property. This distinction is important because many earlier studies focused mainly on mechanical properties or machining responses, while fewer studies reported tensile strength, bending response, impact strength, hardness, surface roughness, cutting force, chip deformation, cutting temperature, tool wear, and multi-response optimization in the same study.

**Table 5:** Comparison of mechanical and machining performance of the present Gr–ZnO–SiC–Al-MMC with reported hybrid MMCs in the literature

Composites	Mechanical properties	Machining performance	Optimization technique	Reference
Al6061 (90 wt%) + graphite (4 wt%) + WC (6wt%)	Tensile strength (141.8 MPa), hardness (91 HV)	—	Deep-learning-assisted mechanical-property prediction	64
Al5083 (97 wt%) + TiC and CuO (3 wt%)	Tensile strength (285.6 MPa), hardness (up to 102.3 HV)	—	Powder metallurgy, compaction and sintering parameter study	65
Al-LM6 (94 wt%) + B_4_C + fly ash (∼6 wt%)	—	MRR (21.423 mm^3^ min^−1^), *R*_a_ (3.06 µm)	Taguchi L27 with GRA	67
Al6061 (95.5 wt%) + SiC (2 wt%) + Gr (2.5 wt%)	Hardness (88 Shore A), compressive strength (4.83 MPa)	Coefficient of friction (0.41)	Experimental study	69
Al 7075 (94 wt%) + B_4_C (2 wt%) + RHA (4 wt%)	—	MRR (23.04 g min^−1^), *R*_a_ (0.457 µm)	Modified Taguchi method	68
Al1060 (79 wt%) + GNS (1 wt%) + SiC (20 wt%)	Tensile strength (171.3 MPa), hardness (67.8 HV)	Coefficient of friction (0.41)	Friction stir processing/process-parameter study	12
Al (95 wt%) + Al_2_O_3_ (2.5 wt%) + ZnO (2.5 wt%)	Hardness (50.23 HV), impact energy absorption (11.77 J), flexural (151.94 MPa)	—	Two-step stir casting; no optimization method reported	10
ZnAl_40_Cu_2_ (95.5 wt%) + B_4_C (1.5 wt%), Gr (3 wt%)	Hardness (163 HB), tensile strength (223 MPa)	Improved wear resistance	Mechanical milling; Gr-content optimization	66
Al (90 wt%) + Gr (2 wt%) + ZnO (2 wt%) + SiC (6 wt%)	Tensile strength (201.5 MPa), flexural strength (283.3 MPa), impact strength (43.3 kJ m^−2^), hardness (69.3 HV)	*R*_a_ (1.46 µm), *F*_c_ (137 N), *ξ* (1.7), *T* (284 °C), wear (0.21 mm)	RSM, TOPSIS, PCA	This study

From a mechanical viewpoint, the present composite achieved a tensile strength of 201.5 MPa, flexural strength of 283.3 MPa, impact strength of 43.3 kJ m^−2^, and hardness of 69.3 HV. Its tensile strength exceeds that of Al1060/GNS/SiC reported by Wang *et al.*^12^ The present tensile strength is also higher than that of the Al6061/WC (tungsten carbide)/graphite composite reported by Shivashankar *et al.*,^64^ although that material showed higher hardness. This difference is reasonable because WC is a very hard ceramic reinforcement and significantly improves indentation resistance, while the present C3 composite contains Gr, which can reduce friction but may not increase hardness as strongly as WC. The higher hardness values reported for Al5083/TiC/CuO^65^ and ZnAl_40_Cu_2_/B_4_C/Gr^66^ are also expected because TiC, CuO, and B_4_C can increase resistance to local plastic deformation. Çanakçı and Çelebi^66^ reported that 3 wt% Gr in ZnAl_40_Cu_2_/B_4_C/Gr produced 163 HB hardness and 223 MPa tensile strength, showing that the optimum Gr content can improve both mechanical and tribological performance when dispersion is controlled. Compared with the Al–Al_2_O_3_–ZnO composite reported by Rumi and Rahman,^10^ the present C3 composite shows higher flexural strength, impact strength, and hardness. This improvement is likely related to the presence of SiC as a primary load-bearing ceramic phase and the controlled amount of Gr, which can assist crack deflection and reduce frictional interaction.

The machining comparison further highlights the contribution of the present work. For the selected C3 composite, MQL-assisted turning produced *R*_a_ = 1.46 µm, *F*_c_ = 137 N, *ξ* = 1.7, *T* = 284 °C, and flank wear of 0.21 mm. This *R*_a_ is lower than that reported for LM6/B_4_C/fly ash WEDM machining.^67^ Prakash *et al.*^67^ used Taguchi L27 and GRA to optimize WEDM of LM6/B_4_C/fly ash hybrid composites for maximum MRR and minimum *R*_a_. The difference in *R*_a_ values should not be interpreted solely as a measure of material superiority, because the compared studies used non-conventional machining processes, different matrices, different reinforcement systems, and different optimization objectives. Instead, the comparison indicates that the present turning-MQL strategy can produce competitive surface quality while monitoring cutting force, chip reduction coefficient, temperature, and wear. The Al7075/B_4_C/RHA study^68^ reported a lower *R*_a_ of 0.58 µm and higher MRR than in the present work; however, it optimized a different Al7075 hybrid system and focused mainly on machining outputs rather than on combined mechanical and machining performance. Similarly, the Al6061/SiC/Gr study^69^ reported hardness, compressive strength, and coefficient of friction, but did not provide a full set of machining responses comparable to the present work. These differences show that direct one-to-one comparisons are limited because reported results depend significantly on the base alloy, reinforcement fraction, fabrication route, machining process, tool material, lubrication condition, and selected objective functions. Recent reviews of hybrid Al-MMCs also emphasize that the reinforcement type, dispersion quality, interfacial bonding, processing route, and machining conditions jointly determine the final mechanical and tribological responses.^70,71^

Overall, Table 5 confirms that the present Gr–ZnO–SiC/Al-MMC offers a competitive, more integrated performance profile than existing hybrid composites. Although some systems show higher hardness, tensile strength, MRR, or lower surface roughness in isolation, this study provides a broader evaluation by combining mechanical characterization, dry and MQL machining assessment, tool-wear analysis, and RSM-TOPSIS-PCA optimization. The main advantage of the C3 composite is its balanced strength, impact resistance, surface quality, reduced cutting force, thermal control, chip flow behavior, and tool-wear mitigation under MQL-assisted turning. This integrated mechanical-machining framework enhances the practical relevance of the proposed tri-hybrid reinforcement strategy for lightweight structural components that require both adequate load-bearing capacity and acceptable machinability.

#### 3.3.7 RSM-based machining parameters optimization

Based on the RSM design described in Section 2.4.2, second-order regression models were developed for the machining responses of the selected C3 composite under dry and MQL turning conditions. The measured responses covered approximately *R*_a_ = 1.31–3.65 µm, *F*_c_ = 95–262 N, *ξ* = 1.68–2.13, and *T* = 239–355 °C, confirming that the selected machining range captured substantial variation in surface quality, cutting load, chip deformation, and thermal response. The fitted environment-specific regression equations are presented in eqn (2)–(9).

2 *R*_a_ (dry) = −0.496344 − 0.008254*V*_c_ − 23.05752*f* + 6.14434*t* + 12*ft* + 2.0 × 10^−5^*V*_c_^2^ + 50.43492*f*^2^ − 2.23374*t*^2^

3 *R*_a_ (MQL) = 1.149623893 − 0.007564818*V*_c_ + 11.84956508*f* − 1.174162783*t* − 5(*f*·*t*) + 2.03889978 × 10^−5^*V*_c_^2^ − 9.143626385*f*^2^ + 1.228331780*t*^2^

4 *F*_c_ (dry) = −260.3293423 + 0.409961686*V*_c_ + 5007.487229*f* − 84.83333333*t* − 4.022988506(*V*_c_·*f*) + 1283.333333(*f*·*t*) − 17407.40741*f*^2^

5 *F*_c_ (MQL) = 180.8324439 + 1.844827586*V*_c_ − 2195.080733*f* − 154.8333333*t* − 15.70881226(*V*_c_·*f*) + 1283.333333(*f*·*t*) + 13730.15873*f*^2^

6 *ξ* (dry) = 3.443752368 + 0.005478927*V*_c_ + 2.179118774*f* − 2.431428571*t* − 0.049808429(*V*_c_·*f*) + 0.868571429*t*^2^

7 *ξ* (MQL) = 3.361444676 + 0.005478927*V*_c_ + 2.179118774*f* − 2.431428571*t* − 0.049808429(*V*_c_·*f*) + 0.868571429*t*^2^

8 *T* (dry) = 115.0114184 + 0.115446005*V*_c_ + 764.5645646*f* + 82.71512892*t* + 5.555555556(*V*_c_·*f*) − 0.896551724(*V*_c_·*t*) − 166.6666667(*f*·*t*) + 0.004170639*V*_c_^2^ − 4009.009009*f*^2^ + 30.27027027*t*^2^

9 *T* (MQL) = 245.5648824 − 0.836238141*V*_c_ + 1739.805323*f* − 177.3072383*t* − 3.065134100(*V*_c_·*f*) + 0.183908046(*V*_c_·*t*) + 3.623767952 × 10^−12^(*f*·*t*) + 0.006534477*V*_c_^2^ − 4594.594595*f*^2^ + 77.83783784*t*^2^

The ANOVA summary in Table 6 confirms that all four global models are significant and that the main effects of *V*_c_, *f*, *t*, and cutting environment govern the machining responses. For *R*_a_, the significant environment-linked term AD, together with the quadratic terms *A*^2^, *B*^2^, and *C*^2^, indicates that surface formation is controlled not only by the direct effects of cutting speed, feed, and depth of cut, but also by the frictional regime imposed by dry or MQL cutting. This agrees with the experimental trend in Fig. 10(a), where MQL consistently lowers *R*_a_. For *F*_c_, several interaction terms, including *AB*, *AD*, *BC*, *BD*, *CD*, and *ABD*, are significant, showing that *F*_c_ is highly sensitive to combined changes in chip load, tool–work contact, and lubrication condition. This is expected because *F*_c_ depends on uncut chip area, shear deformation, particle–tool interaction, and rake-face friction. For *ξ*, fewer interaction terms are significant, suggesting that chip compression is governed mainly by the main cutting variables and the quadratic depth-of-cut effect rather than by extensive higher-order interactions. For *T*, the significant terms *AC*, *AD*, *CD*, *ABD*, *ACD*, *A*^2^, *B*^2^, *C*^2^, and *A*^2^*D* indicate a strongly nonlinear thermal response, which is reasonable because temperature depends on the coupled effects of deformation rate, contact length, friction, and lubrication-assisted heat removal.

**Table 6:** Significance of RSM model terms for machining responses of the selected C3 Al-MMC^a^

Source	*R*_a_	*F*_c_	*ξ*	*T*
Model	✓	✓	✓	✓
*A*	✓	✓	✓	✓
*B*	✓	✓	✓	✓
*C*	✓	✓	✓	✓
*D*	✓	✓	✓	✓
*AB*	✗	✓	✓	✗
*AC*	✗	✗	✗	✓
*AD*	✓	✓	✗	✓
*BC*	✓	✓	✗	✗
*BD*	✓	✓	✗	✗
*CD*	✓	✓	✗	✓
*ABD*	✗	✓	✗	✓
*ACD*	✗	✗	✗	✓
*BCD*	✓	✗	✗	✗
*A* ^2^	✓	✗	✗	✓
*B* ^2^	✓	✓	✗	✓
*C* ^2^	✓	✗	✓	✓
*D* ^2^	✗	✓	✗	✗
*A* ^2^ *D*	✗	✗	✗	✓
*B* ^2^ *D*	✓	✓	✗	✗
*C* ^2^ *D*	✓	✗	✗	✗
Lack of fit	✗	✗	✗	✗

a *A*, *B*, *C*, and *D* denote cutting speed (*V*_c_), feed rate (*f*), depth of cut (*t*), and cutting environment, respectively. ✓ indicates a statistically significant term, and ✗ indicates a statistically insignificant term at the selected confidence level (95%).

The insignificant lack-of-fit values for all four responses indicate that the selected regression forms adequately describe the experimental data within the investigated parameter range. Table 7 further supports the model adequacy. The coefficients of determination are very high for *R*_a_, *F*_c_, and *T* (*R*^2^ = 0.9991, 0.9886, and 0.9995, respectively) and acceptable for *ξ* (*R*^2^ = 0.9225). The predicted *R*^2^ values also remain high, particularly for *R*_a_ and *F*_c_, confirming useful predictive capability. The comparatively lower predicted *R*^2^ for *T* relative to its *R*^2^ may highlight the strong nonlinearity and environment dependence of heat generation and heat removal during turning. This does not invalidate the model, because the prediction error for *T* remains very low, with a MAPE of only 0.18%. Overall, the low MAPE values of 0.82% for *R*_a_, 2.68% for *F*_c_, 1.27% for *ξ*, and 0.18% for *T* show that the fitted models can reliably estimate machining responses. In regression-based machining studies, *R*^2^ values closer to unity are generally preferred because they indicate lower residual error and better model fit.^72^

**Table 7:** Goodness-of-fit and prediction-error statistics for RSM models of machining responses^a^

Model	*R* ^2^	Adjusted *R*^2^	Predicted *R*^2^	Adequate precision	MAPE (%)
*R*_a_	0.9991	0.9981	0.9929	101.8451	0.82
*F*_c_	0.9886	0.9781	0.9178	35.5137	2.68
*ξ*	0.9225	0.8981	0.8343	23.2106	1.27
*T*	0.9995	0.9980	0.8698	109.4488	0.18

a *R* ^2^, adjusted *R*^2^, predicted *R*^2^, adequate precision, and MAPE are used to evaluate model fit, predictive capability, signal-to-noise ratio, and prediction error, respectively.

The diagnostic plots in Fig. 12–14 provide additional validation. The normal probability plots in Fig. 12 show that the externally studentized residuals are closely aligned with the reference line for all responses, indicating approximate normality and the absence of severe outliers. Fig. 13 shows that the residuals are randomly distributed around zero with no visible drift, cyclic pattern, or clustering across the run order. This confirms that the experimental sequence did not introduce systematic bias and that the residuals exhibited reasonably stable variance across the runs. Fig. 14 shows close agreement between experimental and predicted values for *R*_a_, *F*_c_, *ξ*, and *T*. The tightest clustering occurs for *R*_a_ and *T*, consistent with their high *R*^2^ values and low MAPE. The slightly wider scatter in *F*_c_ and *ξ* is expected because these responses are more sensitive to local particle–tool interaction, reinforcement distribution, chip segmentation, and small variations in tool–chip contact during the turning of hybrid Al-MMCs. This explanation is consistent with a recent study showing that the heterogeneous, abrasive nature of MMCs can lead to variable cutting dynamics, surface defects, and tool wear during machining.^18^

**Fig. 12:**
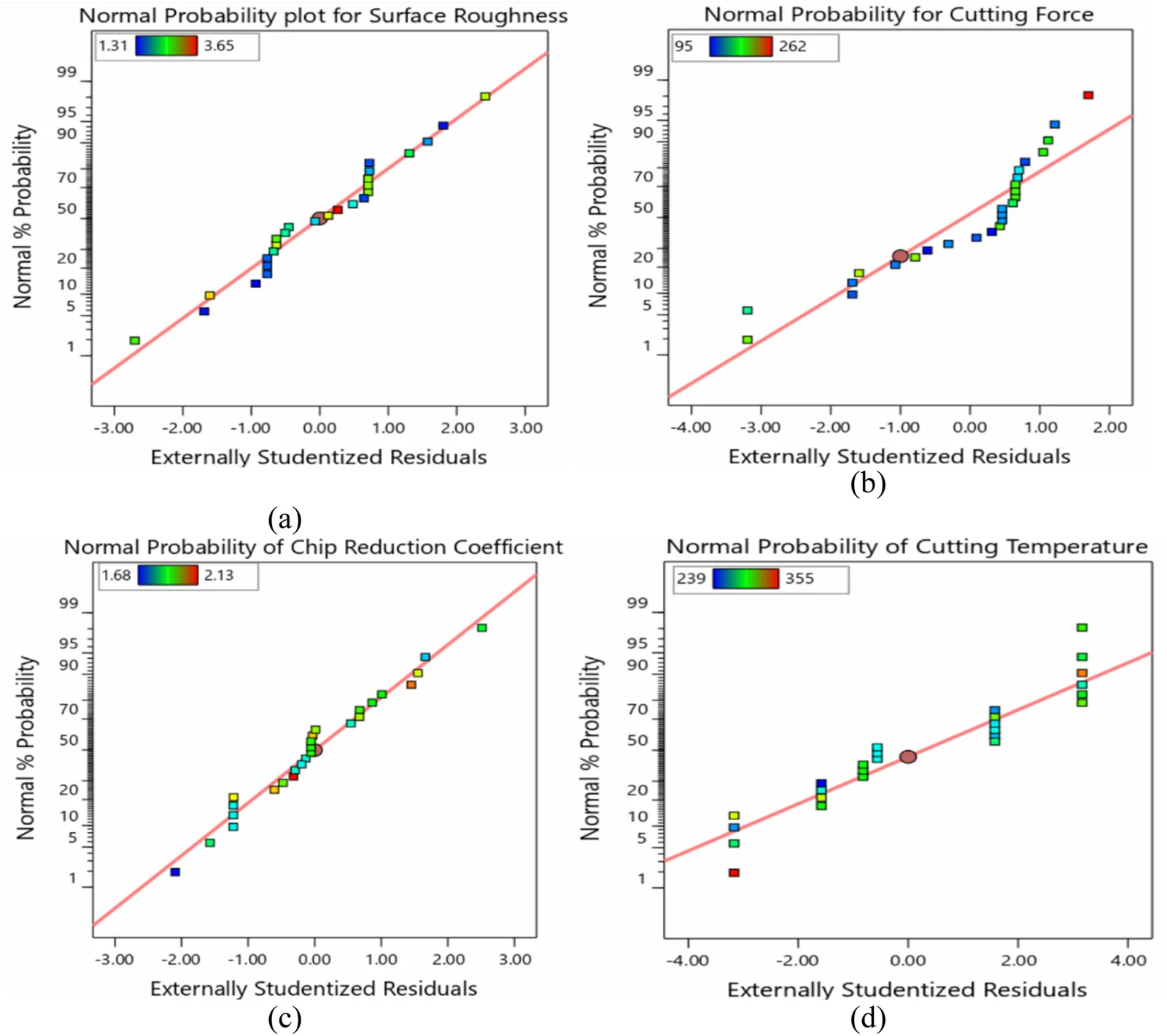
Normal probability plots of externally studentized residuals for the RSM models: (a) surface roughness, (b) cutting force, (c) chip reduction coefficient, and (d) cutting temperature.

**Fig. 13:**
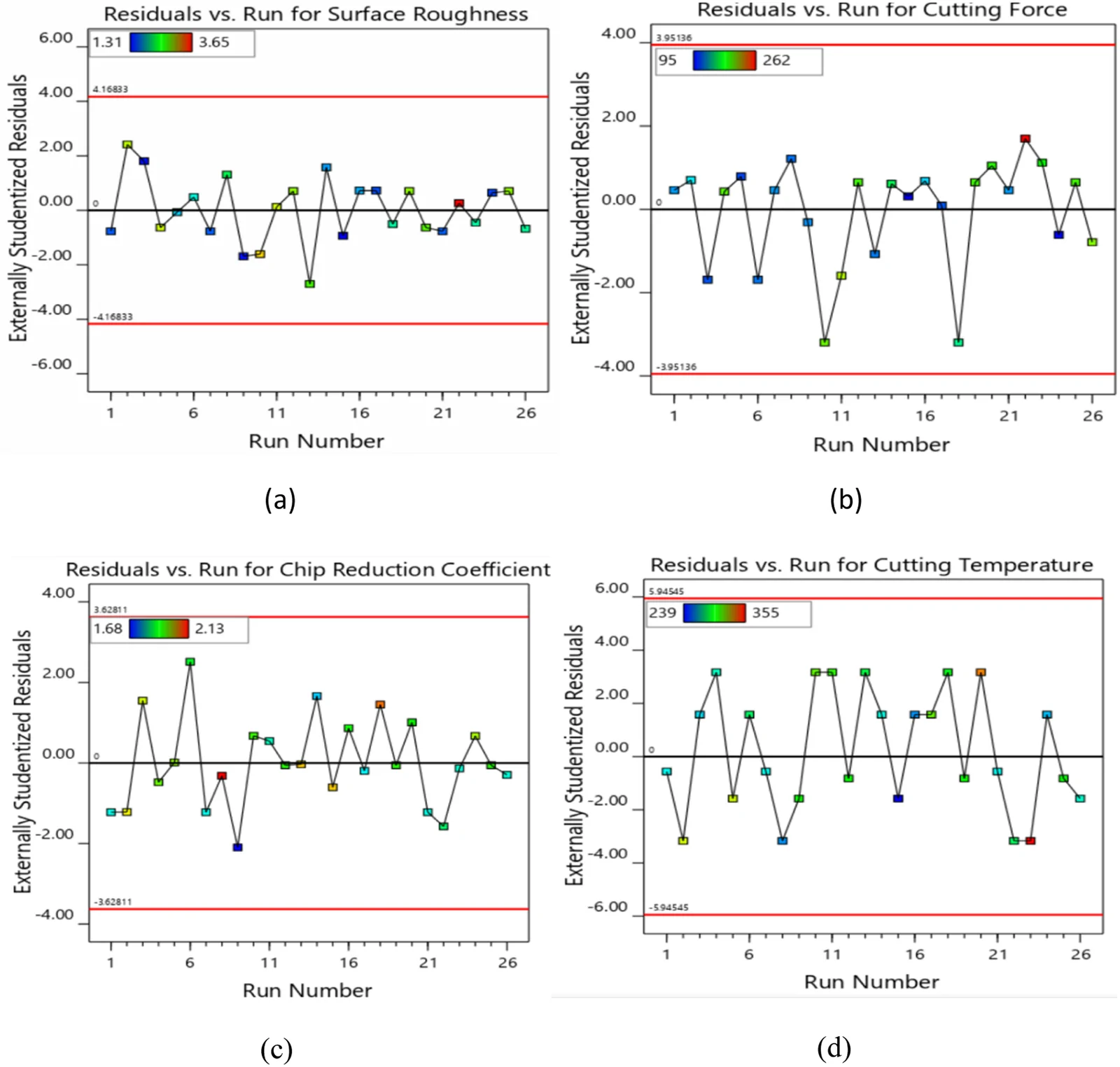
Externally studentized residuals *versus* run order for the RSM models: (a) surface roughness, (b) cutting force, (c) chip reduction coefficient, and (d) cutting temperature.

**Fig. 14:**
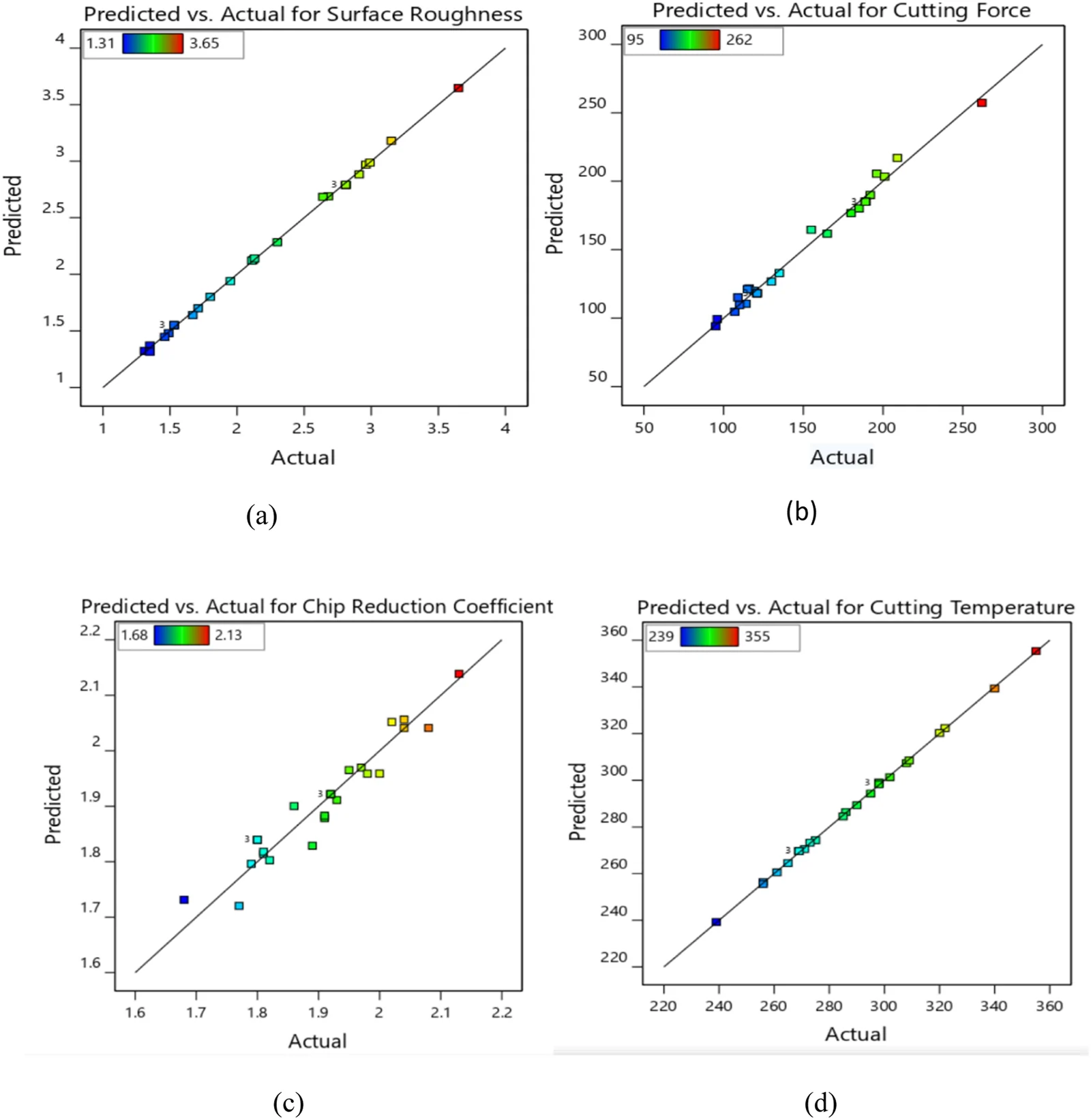
Comparison between experimental and RSM-predicted values for: (a) surface roughness, (b) cutting force, (c) chip reduction coefficient, and (d) cutting temperature.

As presented in Table 8, the best solutions converged within a narrow MQL-assisted operating range near *V*_c_ = 150 m min^−1^, *f* = 0.16 mm per rev, and *t* = 1 mm. The best-ranked solution gives a desirability of 0.897, with predicted responses of *R*_a_ = 1.468 µm, *F*_c_ = 137.604 N, *ξ* = 1.702, and *T* = 284.275 °C. The top seven solutions produced nearly identical desirability values, which indicates that the optimum is not an isolated point but a stable region. This is important for practical machining because small variations around *V*_c_ ≈ 150 m min^−1^ are unlikely to cause major performance deterioration. The optimum combination also aligns with the earlier experimental trends: MQL reduces friction and temperature; higher cutting speed improves shearing and surface formation; higher feed supports productivity; and a lower depth of cut limits cutting load and heat generation. In contrast, the lower-ranked dry solution at *V*_c_ = 91.76 m min^−1^, *f* = 0.10 mm per rev, and *t* = 1 mm produced a much lower desirability of 0.471, confirming that dry cutting cannot provide the same balanced response as MQL for the selected C3 hybrid Al-MMC.

**Table 8:** Multi-response desirability optimization results for turning of the selected C3 Al-MMC

	Input				Responses				
	*A*	*B*	*C*	*D*	*R*_a_	*F*_c_	*ξ*	*T*	Desirability
**1**	**149.89**	**0.16**	**1**	**MQL**	**1.468**	**137.604**	**1.702**	**284.275**	**0.897**
2	150.21	0.16	1	MQL	1.466	137.339	1.701	284.518	0.897
3	149.56	0.16	1	MQL	1.469	137.87	1.702	284.028	0.897
4	150.11	0.16	1	MQL	1.469	137.563	1.7	284.516	0.897
5	150.44	0.16	1	MQL	1.468	137.329	1.699	284.789	0.897
6	149.16	0.16	1	MQL	1.47	138.166	1.703	283.702	0.897
7	149.48	0.16	1	MQL	1.466	137.71	1.704	283.85	0.897
8	149.59	0.16	1	MQL	1.473	138.095	1.7	284.172	0.887
9	150.09	0.16	1	MQL	1.463	137.169	1.704	284.299	0.887
10	148.75	0.16	1	MQL	1.47	138.393	1.704	283.34	0.887
…	…	…	…	…	…	…	…	…	…
47	91.76	0.10	1	Dry	2.753	120.16	2.034	273.384	0.471

Overall, the RSM, ANOVA, residual diagnostics, and desirability results confirm that MQL-assisted turning at approximately *V*_c_ = 150 m min^−1^, *f* = 0.16 mm per rev, and *t* = 1 mm provides the most balanced machining condition for the C3 Al-MMC within the tested domain. The optimized solution is consistent with the measured reductions in surface roughness, cutting force, chip compression, cutting temperature, and flank wear under MQL.

## 4 Conclusions

This study demonstrates a composition-controlled strategy for developing mechanically balanced and machinable Gr–ZnO–SiC–reinforced Al-MMCs *via* multi-stage stir casting. By fixing the Al matrix at 90 wt% and the total reinforcement content at 10 wt%, the work isolates the effect of reinforcement partitioning and shows that the relative fractions of SiC, ZnO, and Gr govern the combined mechanical and machining performance of the composites. Among the investigated compositions, C3, containing 90 wt% Al, 6 wt% SiC, 2 wt% ZnO, and 2 wt% Gr, exhibited the best overall mechanical response. This composite achieved a tensile strength of 201.5 MPa, flexural strength of 283.3 MPa, impact strength of 43.3 kJ m^−2^, and hardness of 69.3 HV. Although C2 showed the highest hardness due to its higher SiC content, C3 provided the most favorable balance among strength, hardness, and impact resistance. The agreement between TOPSIS and PCA identifies C3 as the most balanced multi-response composition. Its superior performance is consistent with SiC-mediated load bearing, Gr-assisted stress transfer and crack deflection, ZnO-related strengthening, and favorable matrix–reinforcement contact. SEM and fractographic observations further showed a compact matrix, no severe agglomeration or large casting voids at the examined locations, and mixed ductile and quasi-cleavage fracture features, consistent with effective load transfer and local matrix plasticity before fracture.

The machining results further confirm that the selected C3 composite is compatible with MQL-assisted turning. Compared with dry cutting, MQL reduced average surface roughness by 43.31%, cutting force by 24.19%, cutting temperature by 8.62%, and chip reduction coefficient by 3.62%. It also reduced flank wear from 0.31 mm under dry cutting to 0.21 mm after 30 min of machining. These improvements are attributed to reduced tool-chip friction, lower adhesion, improved chip flow, suppression of built-up edge formation, and reduced abrasive interaction at the tool-workpiece interface. The combined effect of the intrinsic lubricating properties of Gr and the external MQL mist helped offset the machining difficulties associated with hard SiC and ZnO particles.

The RSM models reliably predicted the machining responses of the C3 composite, as confirmed by significant model terms, an insignificant lack of fit, high coefficients of determination, low prediction errors, and satisfactory residual behavior. Multi-response desirability optimization identified a stable MQL-assisted turning condition at approximately *V*_c_ = 150 m min^−1^, *f* = 0.16 mm per rev, and *t* = 1 mm. This condition produced predicted responses of *R*_a_ = 1.468 µm, *F*_c_ = 137.604 N, *ξ* = 1.702, and *T* = 284.275 °C, with an overall desirability of 0.897. The convergence of several near-identical optimal solutions indicates that the selected condition represents a practical operating region rather than a narrow, isolated optimum.

Overall, this study shows that a carefully balanced Gr–ZnO–SiC reinforcement system can produce an Al-MMC with a strong combination of load-bearing capacity, damage tolerance, surface quality, reduced cutting load, controlled heat generation, and reduced flank wear under MQL machining. The integrated use of mechanical testing, SEM analysis, TOPSIS, PCA, RSM, ANOVA, and desirability optimization provides a rigorous framework for selecting composites and optimizing machining parameters simultaneously. The proposed C3 composite and its optimized MQL turning range offer a promising route for lightweight structural, tribological, automotive, and aerospace components that require both mechanical reliability and sustainable manufacturability. Future work can use SEM-EDS or EPMA/WDS, X-ray micro-CT, XRD/Raman/XPS, HRTEM, and AFM to assess further elemental distribution, porosity, phase constitution, interfacial features, and surface topography. Additional studies may examine fatigue, corrosion, long-term wear, tool-coating effects, advanced cooling, and casting scale-up.

## Author contributions

Md. Sazzad Hossain Ador: conceptualization, methodology, software, formal analysis, investigation, visualization, writing – original draft; Md. Mahraj Uddin: conceptualization, methodology, software, visualization, writing – original draft; Md Zillur Rahman: supervision, validation, project administration, writing – original draft, writing – review & editing.

## Conflicts of interest

The authors declare that they have no known competing financial interests or personal relationships that could have appeared to influence the work reported in this paper.

## Data Availability

All data analyzed during this study are included in this article. No additional datasets were used.
